# Zeolite Adsorption of Chloride from a Synthetic Alkali-Activated Cement Pore Solution

**DOI:** 10.3390/ma12122019

**Published:** 2019-06-24

**Authors:** Jorge Osio-Norgaard, Wil V. Srubar

**Affiliations:** 1 Department of Civil, Environmental, and Architectural Engineering, University of Colorado Boulder, ECOT 441 UCB 428, Boulder, CO 80309-0428, USA; jorge.osionorgaard@colorado.edu; 2 Materials Science and Engineering Program, University of Colorado Boulder, 4001 Discovery Drive, Room N378 80303, Denver CO 80309-0428, USA

**Keywords:** alkali-activated cements, zeolite, faujasite, chabazite, chloride adsorption

## Abstract

This work presents experimental evidence that confirms the potential for two specific zeolites, namely chabazite and faujasite (with a cage size ~2–13 Å), to adsorb small amounts of chloride from a synthetic alkali-activated cement (AAC) pore solution. Four synthetic zeolites were first exposed to a chlorinated AAC pore solution, two faujasite zeolites (i.e., FAU, X-13), chabazite (i.e., SSZ-13), and sodium-stabilized mordenite (i.e., Na-Mordenite). The mineralogy and chemical composition were subsequently investigated via X-ray diffraction (XRD) and both energy- and wavelength-dispersive X-ray spectroscopy (WDS), respectively. Upon exposure to a chlorinated AAC pore solution, FAU and SSZ-13 displayed changes to their diffraction patterns (i.e., peak shifting and broadening), characteristic of ion entrapment within zeolitic aluminosilicate frameworks. Elemental mapping with WDS confirmed the presence of small amounts of elemental chlorine. Results indicate that the chloride-bearing capacity of zeolites is likely dependent on both microstructural features (e.g., cage sizes) and chemical composition.

## 1. Introduction

Given the worldwide prevalence of chloride-induced corrosion in reinforced concrete, the mechanisms of chloride transport and chloride binding in ordinary portland cement (OPC) paste, mortar, and concrete are generally well understood. Chlorides are transported via hydrostatic pressure, capillary absorption, and diffusion [1] and can be bound either as Friedel’s salt in cement paste containing limestone or by hydrotalcite in cement paste containing dolomitic mineral phases [2]. These chloride-binding mechanisms are advantageous, given that they chemically retard the rate of chloride ingress.

Despite previous postulation that zeolites could serve a similar function in alkali-activated cements (AACs), analogous chloride-binding mechanisms in AAC paste, mortar, and concrete have not yet been reported. Zeolites, microporous aluminosilicate minerals found in a variety of low-calcium AACs rich in sodium-stabilized aluminosilicate hydrate (N–A–S–H) gels, have been suggested as prime mineral candidates for chloride adsorption, given their widespread industrial use as molecular sieves, and adsorbents for anions like SO_4_^2−^ and PO_4_^3−^ [1,3,4,5,6]. Recent evidence from Gevaudan et al. [7] and Jun et al. [8] suggest that two specific zeolites—faujasite and chabazite—have the potential to improve concrete chloride resistance (i.e., reduce bulk porosity and enhance chloride binding), thereby delaying the onset of chloride-induced corrosion. However, neither study presented experimental evidence that confirms the potential for these zeolites to physically or chemically adsorb or entrap chlorides in relevant environmental conditions.

The aim of this work was to ascertain whether zeolite minerals commonly found in AAC cement paste, particularly faujasite (FAU), mordenite (MOR), and chabazite, (CHA) are capable of bearing free chlorides. FAU-type zeolites have been observed as reaction products in studies from 2007 to the present [7,9,10,11,12], with [7] discussing the mineralization dynamics of FAU in alkali-activated metakaolin and how the early formation of FAU can lead to a reduced permeable porosity. With regards to the presence of MOR in alkali-activated binders, precursors containing MOR, one of the most common naturally occurring zeolites [13], have been frequently activated and reported in the literature [14,15,16,17,18,19,20,21]. Due to MOR’s particular reticence and poor solubility in high-pH environments [17], a portion of unreacted MOR often remains in the paste. Lastly, CHA-type zeolites have also been identified, studied, and reported in the literature [8,22]. In this study, the synthetic zeolites—two faujasite zeolites that differed only in their respective cage sizes (i.e., FAU, X-13), chabazite (SSZ-13), and sodium-stabilized mordenite (Na-Mordenite)—were exposed to a chlorinated AAC pore solution. While these synthetic zeolites are representative of those found in AAC systems, [7] demonstrated that natural zeolites in AACs systems are meta-stable, especially at early ages, and that their structure can evolve over time into more thermodynamically stable morphologies. Therefore, these synthetic zeolites were chosen to approximate the most likely stable form of these particular zeolites that have been observed to form in AACs. Zeolite mineralogy and chemical composition were subsequently investigated using X-ray diffraction (XRD) and both energy- and wavelength-dispersive X-ray spectroscopy (EDS/WDS), respectively.

## 2. Materials and Methods

### 2.1. Materials

Faujasite zeolites, FAU (cage sizes ~2–13 Å) and X-13 (cage size ~13 Å), were obtained from Sigma Aldrich (St. Louis, MO, USA) and Fisher Scientific (Hampton, NH, USA), respectively. Chabazite zeolites (SSZ-13) were provided by Alfa Aesar (Tewksbury, MA, USA). Na-Mordenite zeolites (MOR) were supplied by Advanced Chemical Supplier (ACS) Material. Sodium hydroxide (NaOH) pellets (97% purity) and powdered sodium chloride (NaCl) (97% purity) were provided by Fisher Scientific (Hampton, NH, USA).

### 2.2. Methods

Using a modified version of exposure conditions found in Ke et al. [21], 1 g of the powdered zeolites was first exposed to 40 mL of the chlorinated synthetic AAC pore solution with a composition of 0.9 M NaOH and 0.1 M NaCl for 14 days under constant agitation. The pore solution composition was chosen based on previously published chloride durability studies [23,24,25] that emphasized the role of alkali hydroxide concentration, while stating that silica, calcium, and aluminum concentrations are typically less than 5 mM and that their role in corrosion are negligible in comparison. Pore solution extractions from multiple AACs show that the concentration of alkali hydroxides is at least two orders of magnitude higher than other constituents [24].

Samples were then centrifuged at 4000 rpm for 6 min, and the supernatant was extracted. Solids were rinsed and filtered with 60 mL of deionized water to remove any salts that may have crystallized on the surface and allowed to dehydrate at ambient conditions.

#### 2.2.1. X-ray Diffraction (XRD)

Qualitative powder XRD analysis of the control and exposed zeolite samples was performed with a Siemens D500 X-ray diffractometer (USGS, Boulder, CO, USA). Samples were analyzed from 5° to 65° 2θ using Cu Kα radiation with a step size of 0.02° and a 2 s dwell time per step. Version 9 of MDI’s Jade XRD software (USGS, Boulder, USA) was used to identify changes in the control and exposed zeolite diffraction patterns [7].

#### 2.2.2. Energy- and Wavelength-Dispersive X-ray Spectroscopy (EDS and WDS)

Using a JEOL-8230 electron microprobe (JEOL, Tokyo, Japan), EDS was utilized to initially screen zeolites for improved chemical mapping. An acceleration voltage of 15 keV and a beam current of 20 nA were used to obtain spectra. With the same instrument, WDS was used to create elemental composition maps of Si Kα, Na Kα, Ca Kα, and Cl Kα. For all samples, two Cl Kα maps were aggregated utilizing National Institute of Health (NIH) ImageJ software (NIH, Bethesda, MD, USA) to obtain better resolution. A similar acceleration voltage of 15 keV, a beam current of 20 nA, and a dwell time of 15 ms were used to obtain all maps. The electron beam was defocused to 0.5 µm, providing a 1:1 ratio between the beam and pixel size. The resulting maps measured approximately 500 µm × 500 µm in area. All raw data are provided in the supplementary material.

## 3. Results

### 3.1. XRD

**Figure 1:**
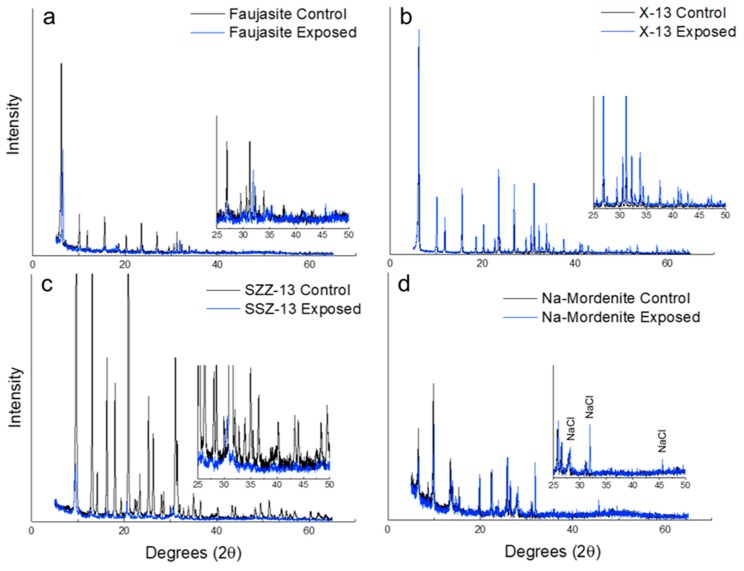
Diffraction patterns of (**a**) faujasite (FAU) (cage size ~2–13 Å), (**b**) faujasite (X-13) (cage size ~13 Å), (**c**) chabazite (SSZ-13), and (**d**) sodium-stabilized mordenite (Na-Mordenite).

Upon exposure to a chloride-laden synthetic AAC pore solution, both FAU and SSZ-13 exhibited evidence of peak broadening, shifting, and intensity reductions in comparison to their respective control diffraction patterns (Figure 1). Crystallographic theory indicates that these shifts can be due to lattice strain, due to the incorporation of ions into zeolitic aluminosilicate frameworks, which has been discussed for both cationic and anionic sorption processes of zeolite [22,26,27]. Therefore, the peak shifting and broadening provided an indication that these zeolites may have adsorbed free ions (e.g., Na, Cl) from the AAC pore solution. However, these diffraction patterns alone were insufficient to confirm whether FAU and SSZ-13 adsorbed or entrapped free Cl rather than Na (or a combination of the two) in their ionic state. X-13 (Figure 1b) and Na-Mordenite (Figure 1d) indicated little to no changes to their diffraction patterns after exposure. Of note, the diffraction pattern for Na-Mordenite was the only zeolite sample to indicate the presence of crystalline NaCl post-exposure.

### 3.2. EDS and WDS

**Figure 2:**
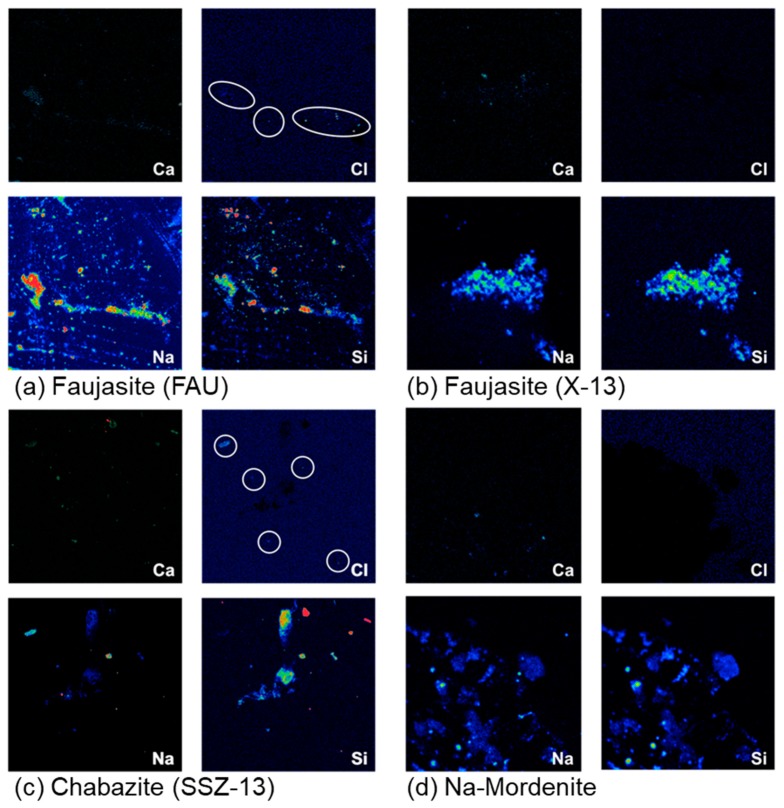
WDS (wavelength-dispersive X-ray spectroscopy) elemental composition maps of chloride-exposed (**a**) faujasite (FAU), (**b**) faujasite (X-13), (**c**) chabazite (SSZ-13), and (**d**) mordenite (Na-Mordenite).

After confirmation via EDS that elemental chlorine was present in FAU and SSZ-13 (Table 1), WDS elemental composition maps were obtained to substantiate the presence of small amounts of free chlorides (Figure 2). Elemental composition maps for Na-Mordenite and X-13 were also generated, but yielded no presence of Cl higher than background levels. Figure 2a,c indicates that chloride is present in some siliceous regions (i.e., regions of high Si) of FAU and SSZ-13. Silicon (Si) maps were used in this study to visualize the geometry of the aluminosilicate zeolites. The Na and Cl maps in Figure 2 suggest that small amounts of elemental chlorine are adsorbed by FAU and SSZ-13 without being bound as NaCl, while Figure 2c does indicate the likelihood of the presence of some NaCl in the SSZ-13 sample. Understandably, this result could be interpreted as the formation of crystalline NaCl in FAU and SSZ-13. However, it is of note that both FAU and SSZ-13 zeolites also contain Na before exposure, and a significant presence of crystalline NaCl would be detected via XRD. The overlap between Si and Na is more prevalent and serves as an indicator that, in this instance, the Na is more than likely part of the original structure of the FAU and SSZ-13 zeolites.

## 4. Discussion

Chloride-mineral interactions (e.g., adsorption, uptake, or binding) have been widely studied in OPC and slag-based AAC systems, but the role of zeolites in chloride transport is widely unexplored [1]. C_3_A in OPC systems and dolomitic minerals in slag cements have both been shown to bind non-trivial quantities of chloride ions upon exposure to chlorinated solutions [2,3,4]. Zeolites are structurally different (e.g., in silica content and charge) than layered double hydroxides and are primarily viewed as cation binders. However [5,6] have shown that they are capable of weak anionic binding. This study is the first to directly assess the chloride uptake potential of these zeolites. While a direct comparison between the extent of chloride binding potential of zeolites versus the calcium aluminate phases in OPC could not be made in this study, future studies should aim to quantify the chloride adsorption potential of zeolites to compare them with the mineral phases in hydrated OPC [2,3,4].

Taken together, XRD, EDS, and WDS substantiate clear evidence that, while some zeolites are capable of bearing chloride, the capability likely depends on microstructure features, such as cage size and chemical composition. FAU and X-13—two faujasite zeolites that differ only in cage size—behaved differently in terms of their potential for chloride adsorption. X-13 has, on average, larger zeolitic cages than faujasite, which may enable chloride ions to flow through more freely. Contrastingly, SSZ-13 has a similar average cage size to X-13 but a different chemical composition (e.g., Si:Al ratio [27]), which indicates that cage size is not the only influencing factor.

## 5. Conclusions

This work presents experimental evidence of chloride-bearing potential for two zeolites—faujasite (FAU) and chabazite (SSZ-13)—commonly found in alkali-activated cement (AAC) paste, mortar, and concrete. Results from X-ray diffraction and both energy- and wavelength dispersive X-ray spectroscopy indicate that these zeolites contain small—but non-trivial—amounts of elemental chlorine that was adsorbed from a chlorinated synthetic AAC pore solution after 14 days of exposure. Another faujasite zeolite (X-13) with a larger average cage size than FAU did not exhibit a similar potential to adsorb chlorides. Taken together, these results suggest that the chloride adsorption potential of zeolites is likely linked to both microstructural features and chemical composition. Further research is needed to elucidate and leverage the explicit chloride-uptake mechanisms in these (and other) zeolites and to quantify the extent to which chloride uptake can be enhanced. These efforts would be impactful, as they would enable the design of more chloride-resistant AAC paste, mortar, and concrete.

## Figures and Tables

**Table 1:** Summary of evidence indicating the chloride-bearing potential of zeolites.

Sample	Zeolite	XRD Peak Shifts or Broadening	XRD NaCl Presence	EDS Chlorine Presence	WDS Chlorine Presence
FAU	Faujasite	Yes	No	Yes	Yes
X-13	Faujasite	No	No	No	No
SSZ-13	Chabazite	Yes	No	Yes	Yes
Na-Mordenite	Mordenite	No	Yes	No	No

## Supplementary Materials

The following is available online at https://www.mdpi.com/1996-1944/12/12/2019/s1: Raw Data Repository.
