# Supplementary figures and images for: Zeolite Adsorption of Chloride from a Synthetic Alkali-Activated Cement Pore Solution
Source: Materials (Basel). 2019 Jun 24;12(12):2019. doi: 10.3390/ma12122019 (PMC6630726; doi:10.3390/ma12122019)

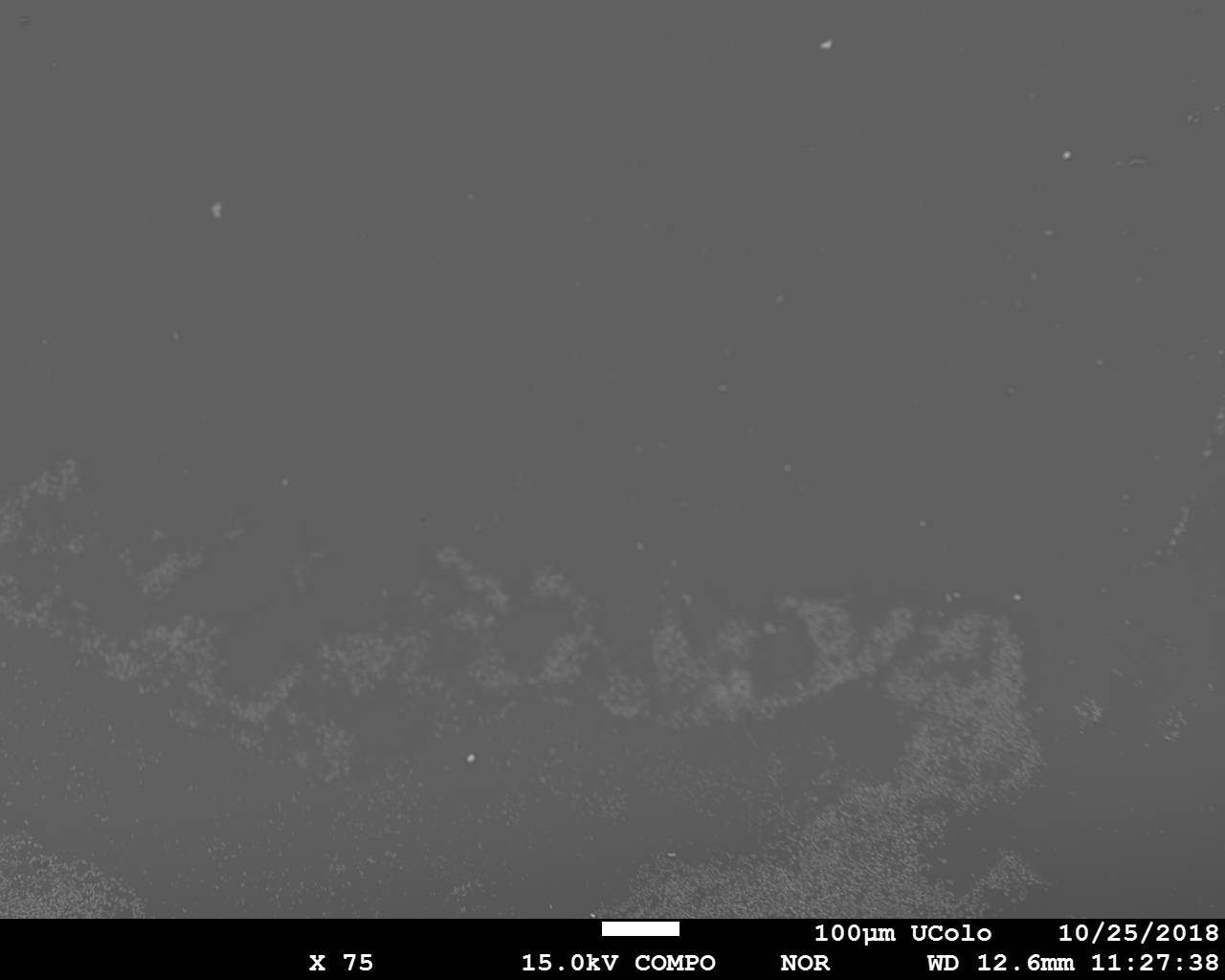

Supplement: Supplementary file 1 [file materials-12-02019-s001.zip › Raw Data for MDPI Repository/FAU (X-13)/X-13 Control.tif]

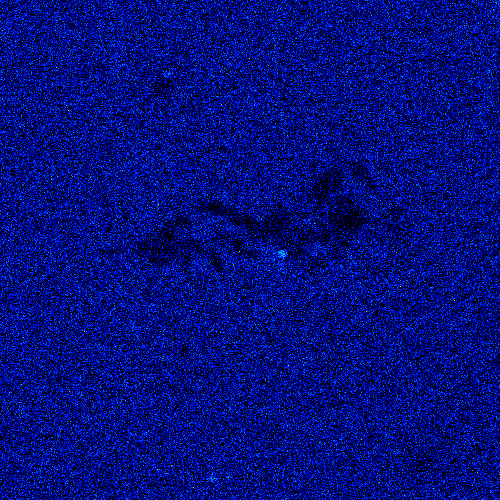

Supplement: Supplementary file 1 [file materials-12-02019-s001.zip › Raw Data for MDPI Repository/FAU (X-13)/(X-13)Zeolite Maps_0001_MAP_0007_image/Cl_2.tif]

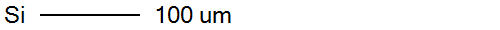

Supplement: Supplementary file 1 [file materials-12-02019-s001.zip › Raw Data for MDPI Repository/FAU (X-13)/(X-13)Zeolite Maps_0001_MAP_0007_image/Si_marker.tif]

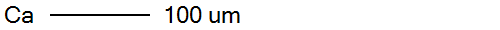

Supplement: Supplementary file 1 [file materials-12-02019-s001.zip › Raw Data for MDPI Repository/FAU (X-13)/(X-13)Zeolite Maps_0001_MAP_0007_image/Ca_marker.tif]

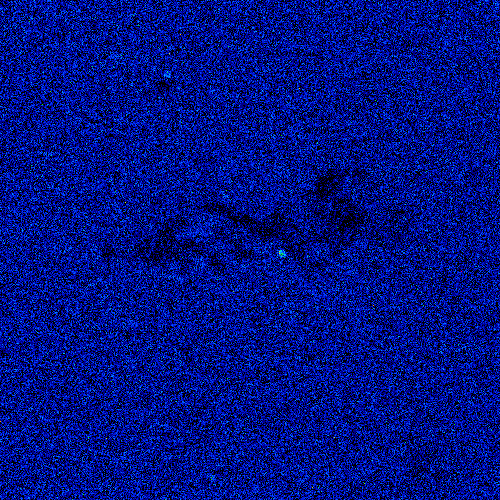

Supplement: Supplementary file 1 [file materials-12-02019-s001.zip › Raw Data for MDPI Repository/FAU (X-13)/(X-13)Zeolite Maps_0001_MAP_0007_image/Cl.tif]

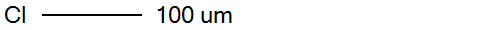

Supplement: Supplementary file 1 [file materials-12-02019-s001.zip › Raw Data for MDPI Repository/FAU (X-13)/(X-13)Zeolite Maps_0001_MAP_0007_image/Cl_marker.tif]

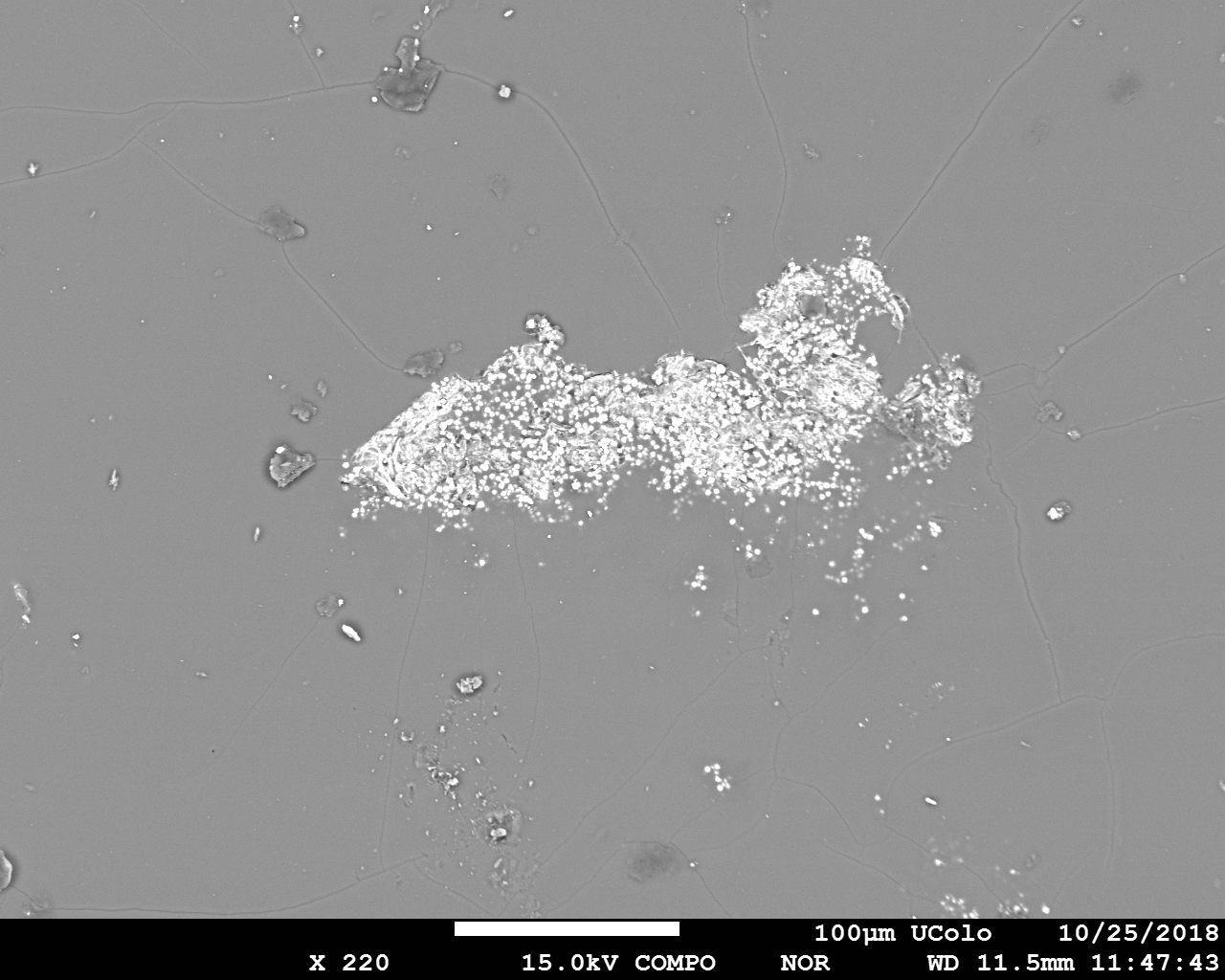

Supplement: Supplementary file 1 [file materials-12-02019-s001.zip › Raw Data for MDPI Repository/FAU (X-13)/(X-13)Zeolite Maps_0001_MAP_0007_image/X-13 Exposed - Copy.tif]

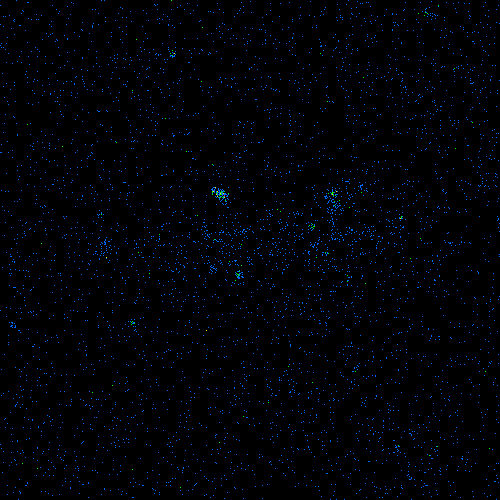

Supplement: Supplementary file 1 [file materials-12-02019-s001.zip › Raw Data for MDPI Repository/FAU (X-13)/(X-13)Zeolite Maps_0001_MAP_0007_image/Ca.tif]

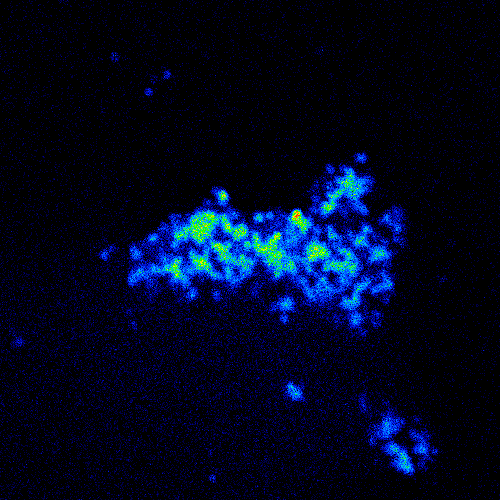

Supplement: Supplementary file 1 [file materials-12-02019-s001.zip › Raw Data for MDPI Repository/FAU (X-13)/(X-13)Zeolite Maps_0001_MAP_0007_image/Na.tif]

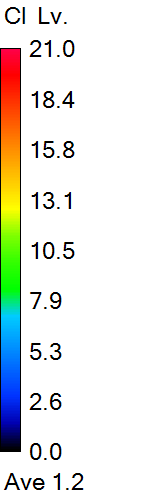

Supplement: Supplementary file 1 [file materials-12-02019-s001.zip › Raw Data for MDPI Repository/FAU (X-13)/(X-13)Zeolite Maps_0001_MAP_0007_image/Cl_2_color.tif]

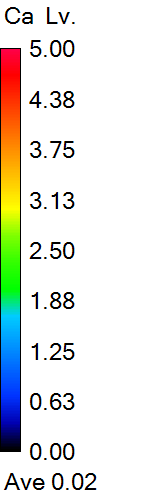

Supplement: Supplementary file 1 [file materials-12-02019-s001.zip › Raw Data for MDPI Repository/FAU (X-13)/(X-13)Zeolite Maps_0001_MAP_0007_image/Ca_color.tif]

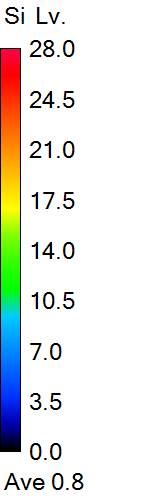

Supplement: Supplementary file 1 [file materials-12-02019-s001.zip › Raw Data for MDPI Repository/FAU (X-13)/(X-13)Zeolite Maps_0001_MAP_0007_image/Si_color.tif]

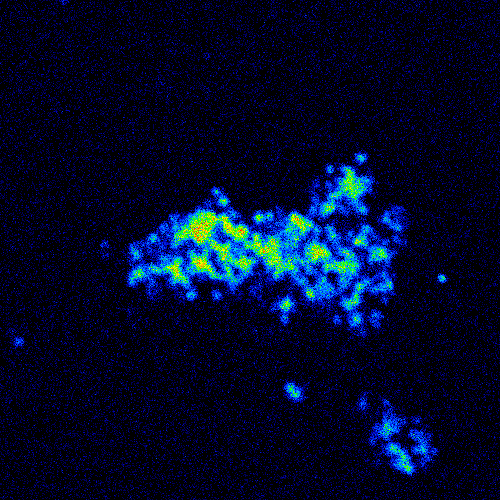

Supplement: Supplementary file 1 [file materials-12-02019-s001.zip › Raw Data for MDPI Repository/FAU (X-13)/(X-13)Zeolite Maps_0001_MAP_0007_image/Si.tif]

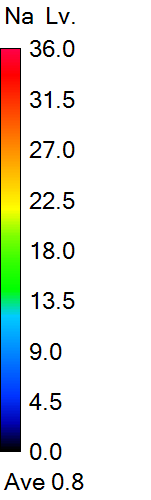

Supplement: Supplementary file 1 [file materials-12-02019-s001.zip › Raw Data for MDPI Repository/FAU (X-13)/(X-13)Zeolite Maps_0001_MAP_0007_image/Na_color.tif]

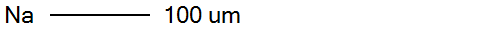

Supplement: Supplementary file 1 [file materials-12-02019-s001.zip › Raw Data for MDPI Repository/FAU (X-13)/(X-13)Zeolite Maps_0001_MAP_0007_image/Na_marker.tif]

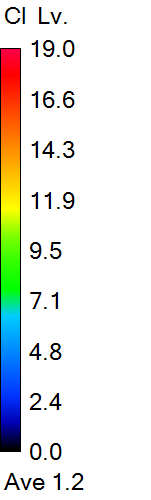

Supplement: Supplementary file 1 [file materials-12-02019-s001.zip › Raw Data for MDPI Repository/FAU (X-13)/(X-13)Zeolite Maps_0001_MAP_0007_image/Cl_color.tif]

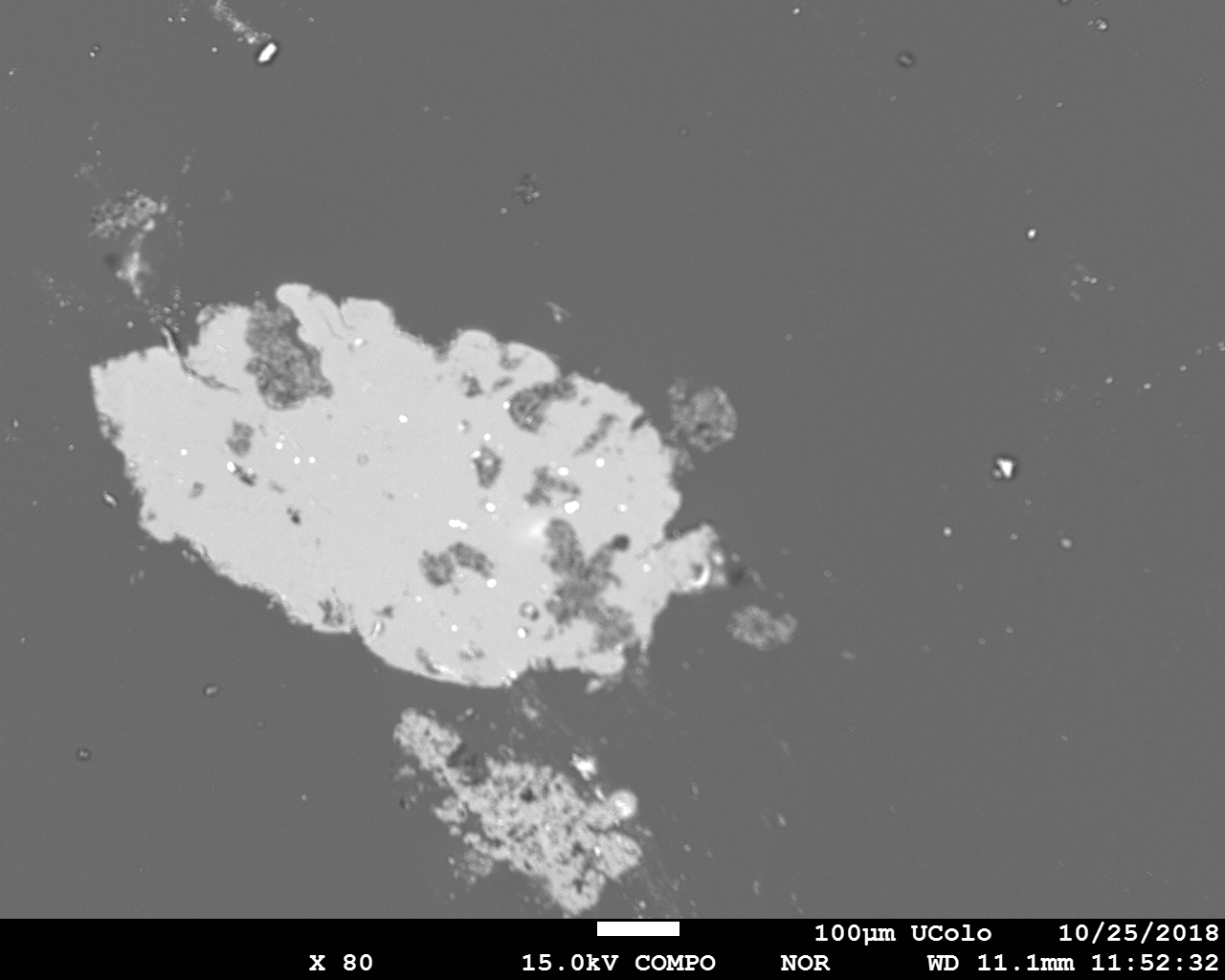

Supplement: Supplementary file 1 [file materials-12-02019-s001.zip › Raw Data for MDPI Repository/MOR/Mordenite Exposed.tif]

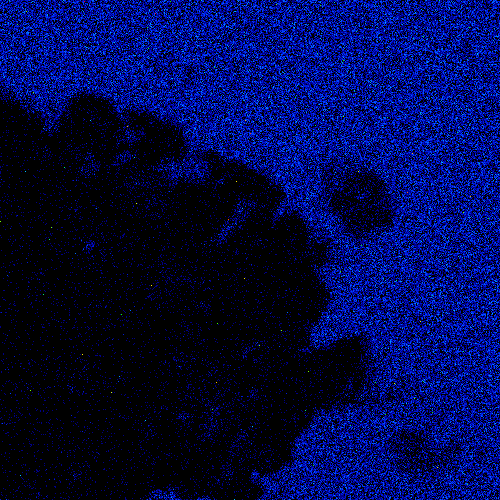

Supplement: Supplementary file 1 [file materials-12-02019-s001.zip › Raw Data for MDPI Repository/MOR/(MORDENITE) Zeolite Maps_0001_MAP_0008_image/Cl_2.tif]

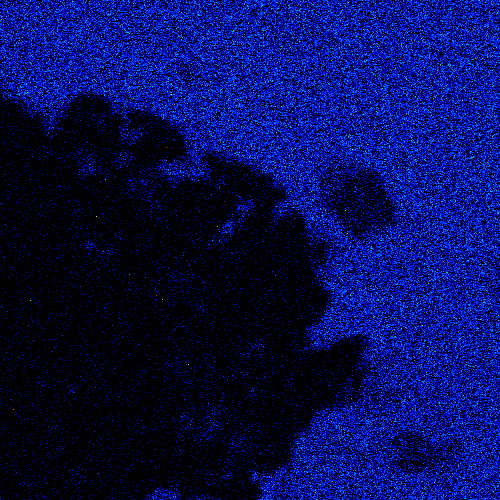

Supplement: Supplementary file 1 [file materials-12-02019-s001.zip › Raw Data for MDPI Repository/MOR/(MORDENITE) Zeolite Maps_0001_MAP_0008_image/Cl.tif]

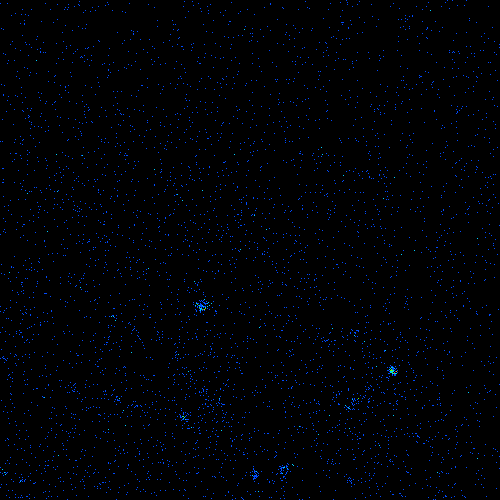

Supplement: Supplementary file 1 [file materials-12-02019-s001.zip › Raw Data for MDPI Repository/MOR/(MORDENITE) Zeolite Maps_0001_MAP_0008_image/Ca.tif]

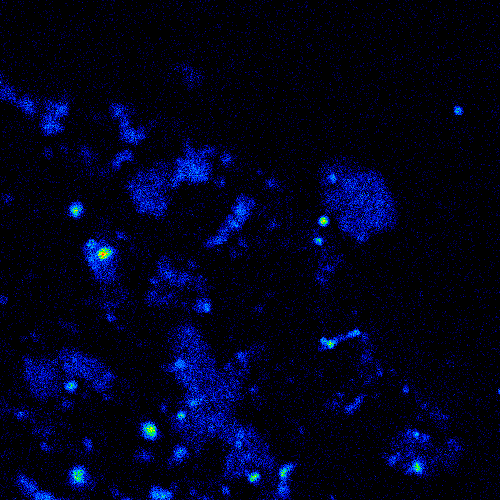

Supplement: Supplementary file 1 [file materials-12-02019-s001.zip › Raw Data for MDPI Repository/MOR/(MORDENITE) Zeolite Maps_0001_MAP_0008_image/Na.tif]

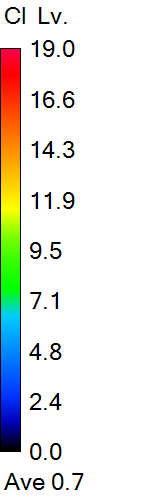

Supplement: Supplementary file 1 [file materials-12-02019-s001.zip › Raw Data for MDPI Repository/MOR/(MORDENITE) Zeolite Maps_0001_MAP_0008_image/Cl_2_color.tif]

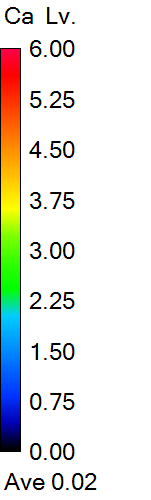

Supplement: Supplementary file 1 [file materials-12-02019-s001.zip › Raw Data for MDPI Repository/MOR/(MORDENITE) Zeolite Maps_0001_MAP_0008_image/Ca_color.tif]

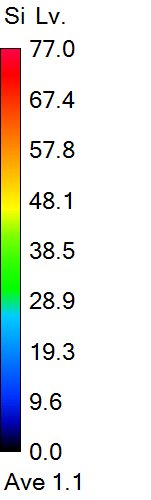

Supplement: Supplementary file 1 [file materials-12-02019-s001.zip › Raw Data for MDPI Repository/MOR/(MORDENITE) Zeolite Maps_0001_MAP_0008_image/Si_color.tif]

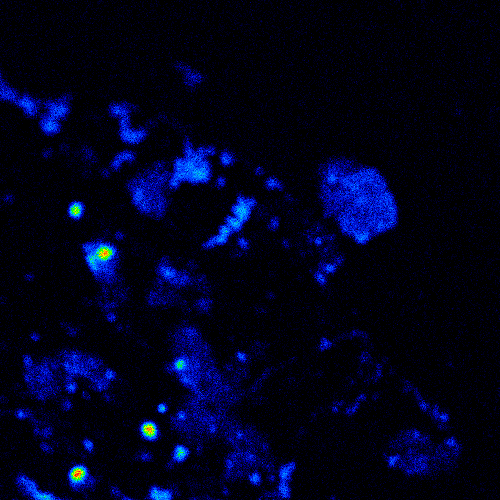

Supplement: Supplementary file 1 [file materials-12-02019-s001.zip › Raw Data for MDPI Repository/MOR/(MORDENITE) Zeolite Maps_0001_MAP_0008_image/Si.tif]

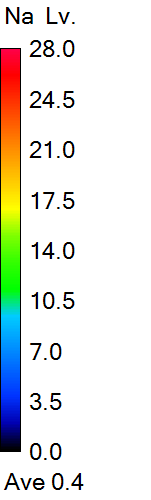

Supplement: Supplementary file 1 [file materials-12-02019-s001.zip › Raw Data for MDPI Repository/MOR/(MORDENITE) Zeolite Maps_0001_MAP_0008_image/Na_color.tif]

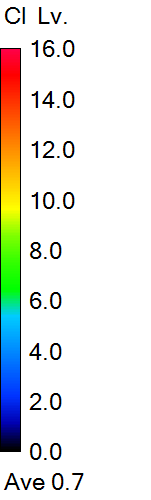

Supplement: Supplementary file 1 [file materials-12-02019-s001.zip › Raw Data for MDPI Repository/MOR/(MORDENITE) Zeolite Maps_0001_MAP_0008_image/Cl_color.tif]

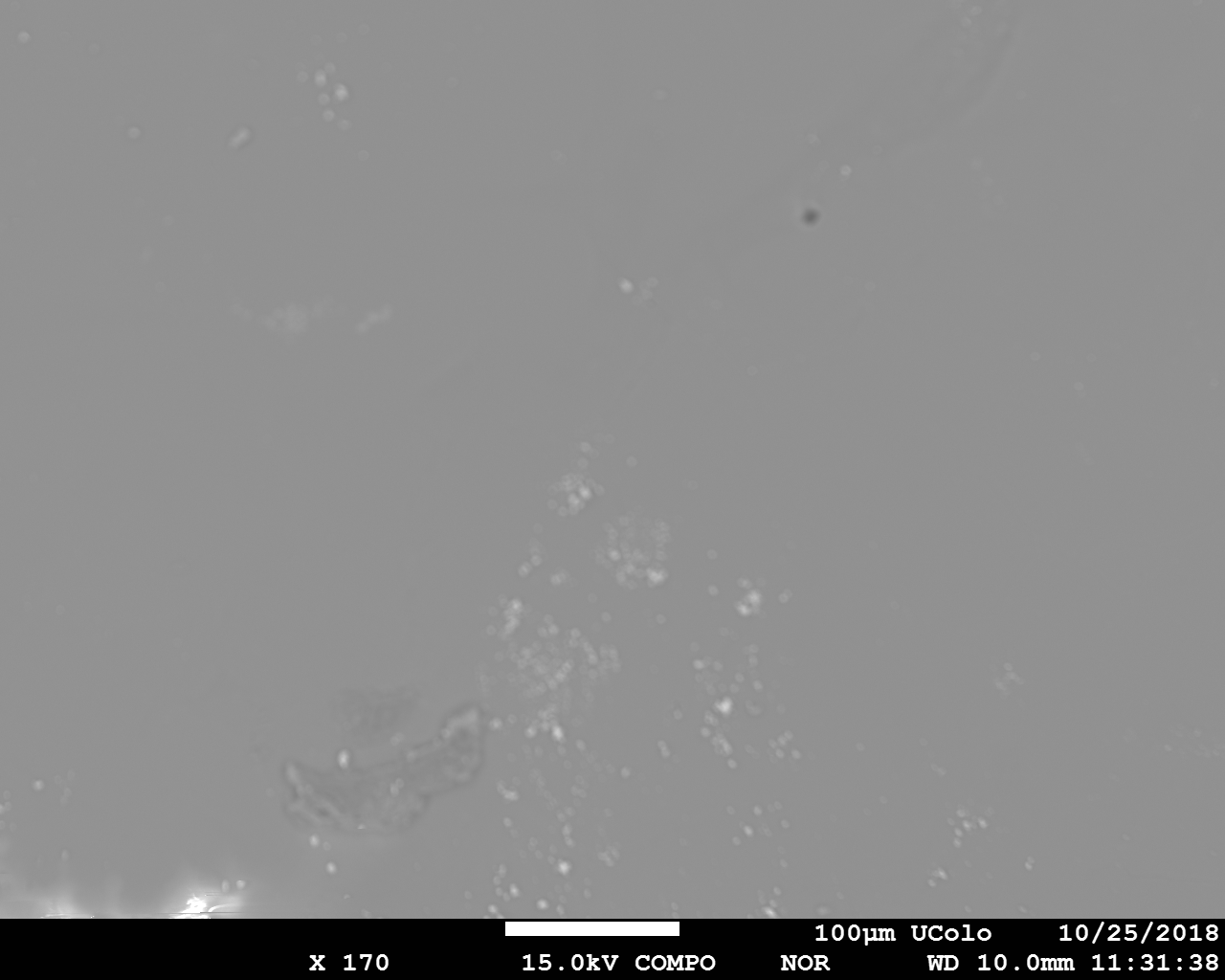

Supplement: Supplementary file 1 [file materials-12-02019-s001.zip › Raw Data for MDPI Repository/MOR/Mordenite Control.tif]

## Slide 1
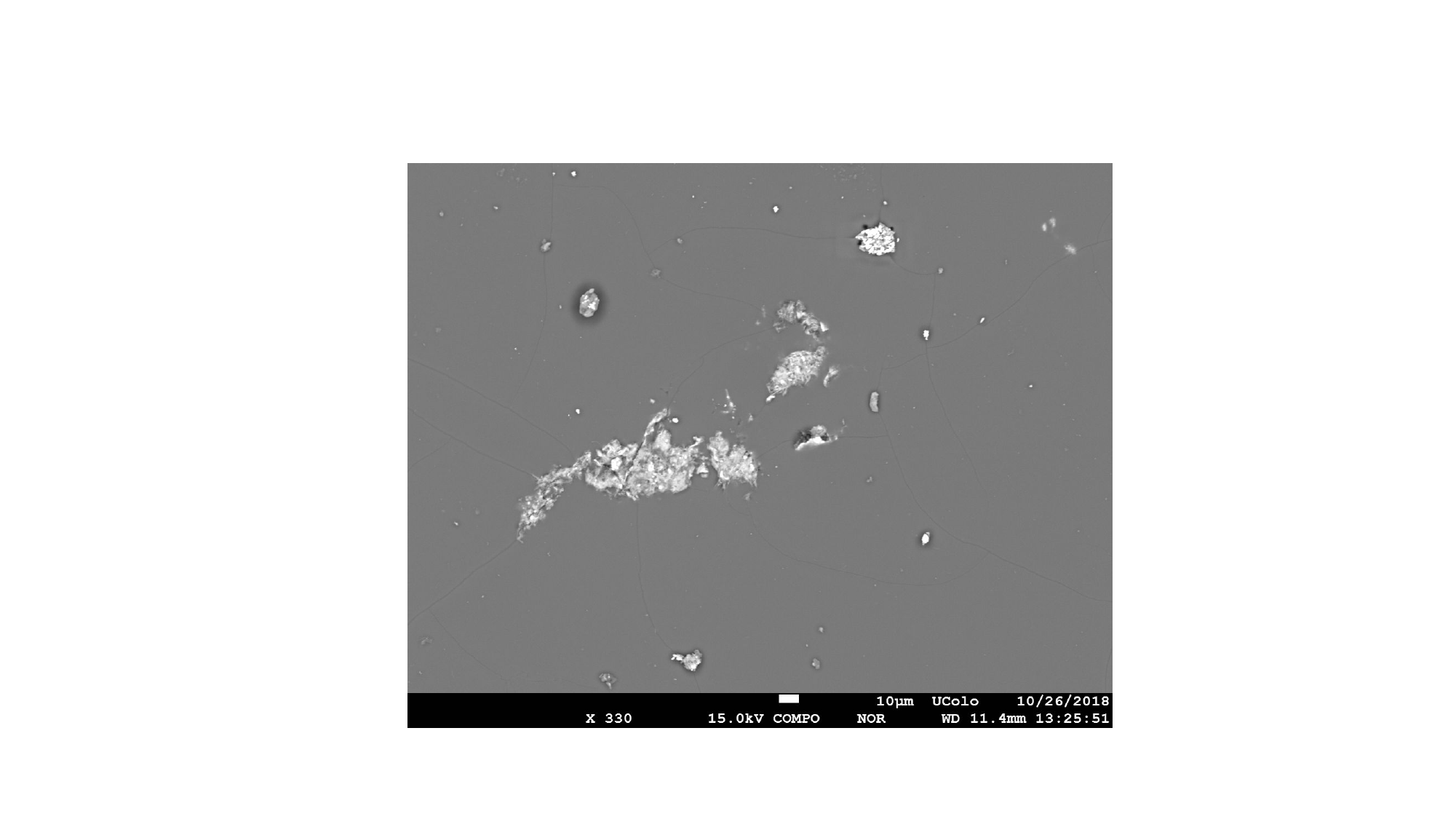

## Slide 2
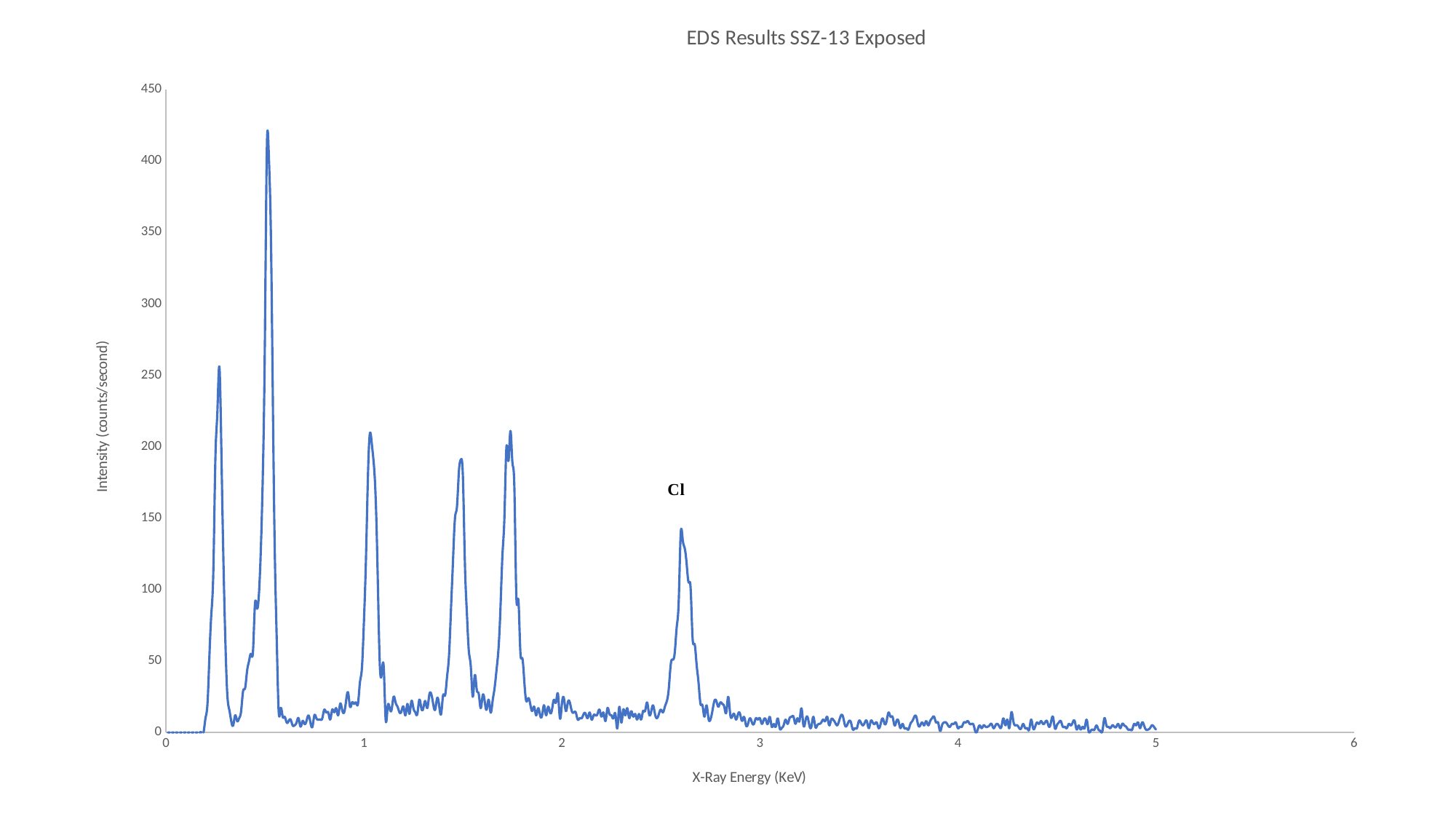

### Chart: EDS Results SSZ-13 Exposed
| Category | |
|---|---|

Supplement: Supplementary file 1 [file materials-12-02019-s001.zip › Raw Data for MDPI Repository/CHA (SSZ-13)/EDS/EDS Results.pptx]

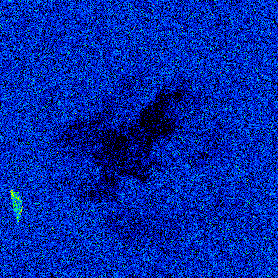

Supplement: Supplementary file 1 [file materials-12-02019-s001.zip › Raw Data for MDPI Repository/CHA (SSZ-13)/SSZ13_3 Exposed/Cl_2.tif]

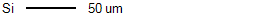

Supplement: Supplementary file 1 [file materials-12-02019-s001.zip › Raw Data for MDPI Repository/CHA (SSZ-13)/SSZ13_3 Exposed/Si_marker.tif]

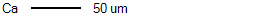

Supplement: Supplementary file 1 [file materials-12-02019-s001.zip › Raw Data for MDPI Repository/CHA (SSZ-13)/SSZ13_3 Exposed/Ca_marker.tif]

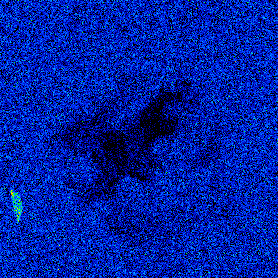

Supplement: Supplementary file 1 [file materials-12-02019-s001.zip › Raw Data for MDPI Repository/CHA (SSZ-13)/SSZ13_3 Exposed/Cl.tif]

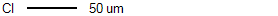

Supplement: Supplementary file 1 [file materials-12-02019-s001.zip › Raw Data for MDPI Repository/CHA (SSZ-13)/SSZ13_3 Exposed/Cl_marker.tif]

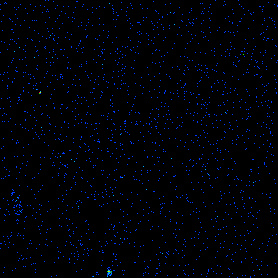

Supplement: Supplementary file 1 [file materials-12-02019-s001.zip › Raw Data for MDPI Repository/CHA (SSZ-13)/SSZ13_3 Exposed/Ca.tif]

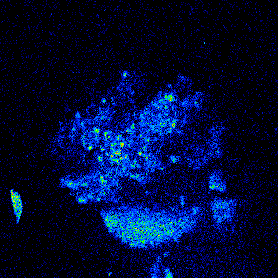

Supplement: Supplementary file 1 [file materials-12-02019-s001.zip › Raw Data for MDPI Repository/CHA (SSZ-13)/SSZ13_3 Exposed/Na.tif]

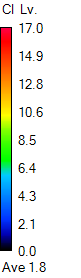

Supplement: Supplementary file 1 [file materials-12-02019-s001.zip › Raw Data for MDPI Repository/CHA (SSZ-13)/SSZ13_3 Exposed/Cl_2_color.tif]

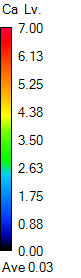

Supplement: Supplementary file 1 [file materials-12-02019-s001.zip › Raw Data for MDPI Repository/CHA (SSZ-13)/SSZ13_3 Exposed/Ca_color.tif]

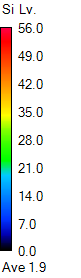

Supplement: Supplementary file 1 [file materials-12-02019-s001.zip › Raw Data for MDPI Repository/CHA (SSZ-13)/SSZ13_3 Exposed/Si_color.tif]

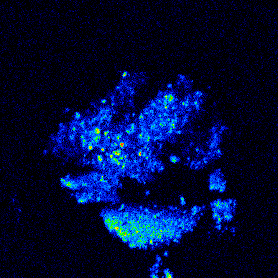

Supplement: Supplementary file 1 [file materials-12-02019-s001.zip › Raw Data for MDPI Repository/CHA (SSZ-13)/SSZ13_3 Exposed/Si.tif]

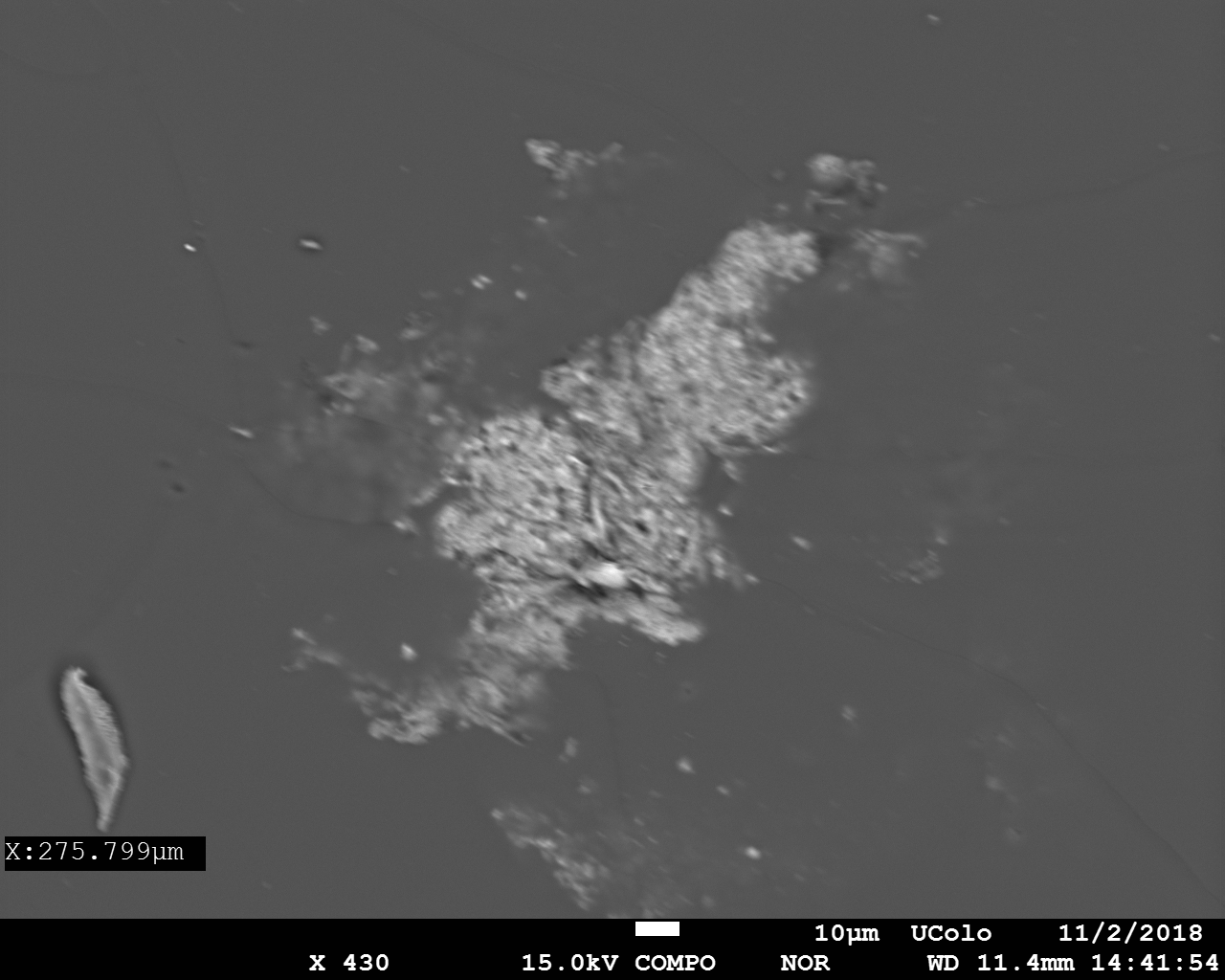

Supplement: Supplementary file 1 [file materials-12-02019-s001.zip › Raw Data for MDPI Repository/CHA (SSZ-13)/SSZ13_3 Exposed/SSZ-13_6.tif]

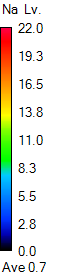

Supplement: Supplementary file 1 [file materials-12-02019-s001.zip › Raw Data for MDPI Repository/CHA (SSZ-13)/SSZ13_3 Exposed/Na_color.tif]

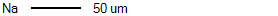

Supplement: Supplementary file 1 [file materials-12-02019-s001.zip › Raw Data for MDPI Repository/CHA (SSZ-13)/SSZ13_3 Exposed/Na_marker.tif]

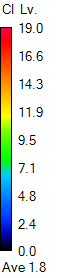

Supplement: Supplementary file 1 [file materials-12-02019-s001.zip › Raw Data for MDPI Repository/CHA (SSZ-13)/SSZ13_3 Exposed/Cl_color.tif]

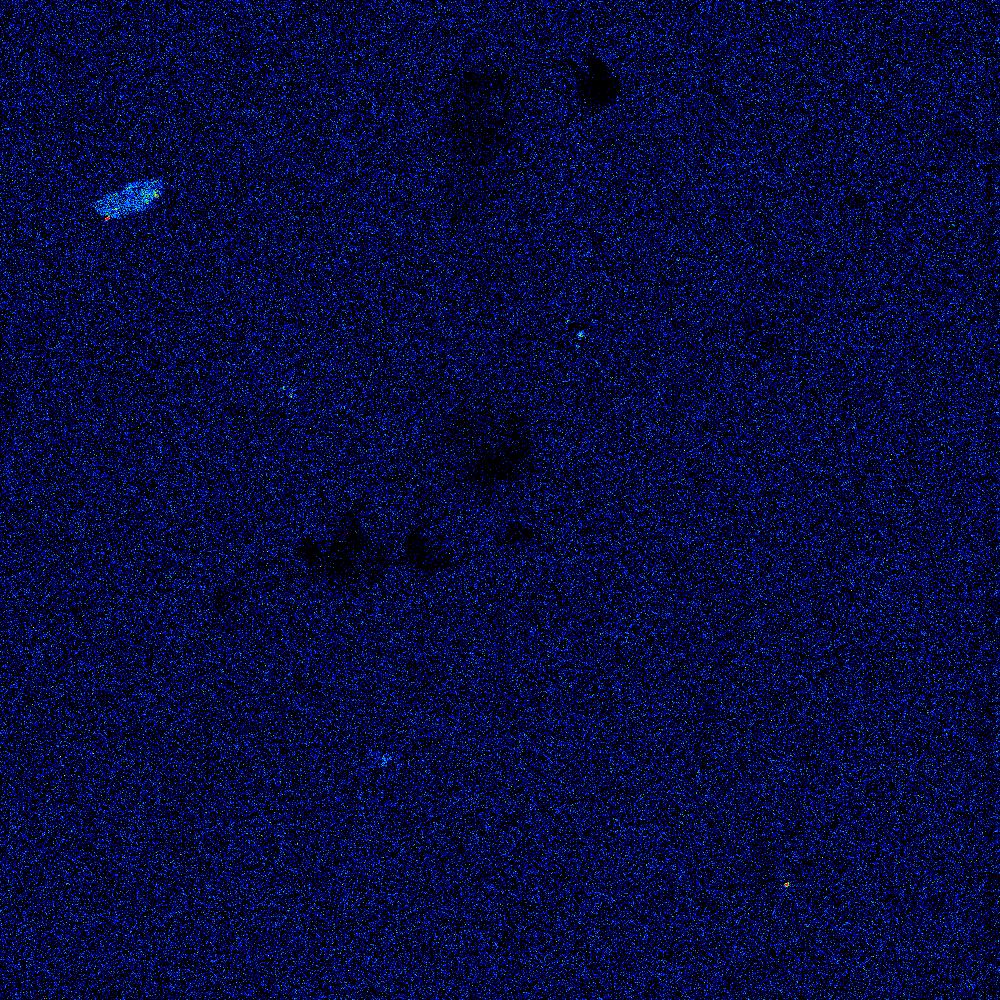

Supplement: Supplementary file 1 [file materials-12-02019-s001.zip › Raw Data for MDPI Repository/CHA (SSZ-13)/WDS/Cl_2.tif]

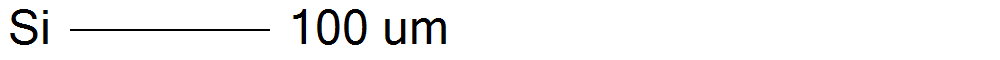

Supplement: Supplementary file 1 [file materials-12-02019-s001.zip › Raw Data for MDPI Repository/CHA (SSZ-13)/WDS/Si_marker.tif]

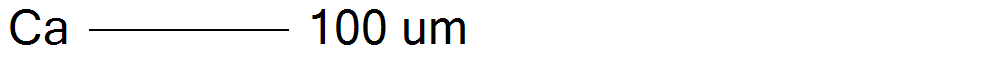

Supplement: Supplementary file 1 [file materials-12-02019-s001.zip › Raw Data for MDPI Repository/CHA (SSZ-13)/WDS/Ca_marker.tif]

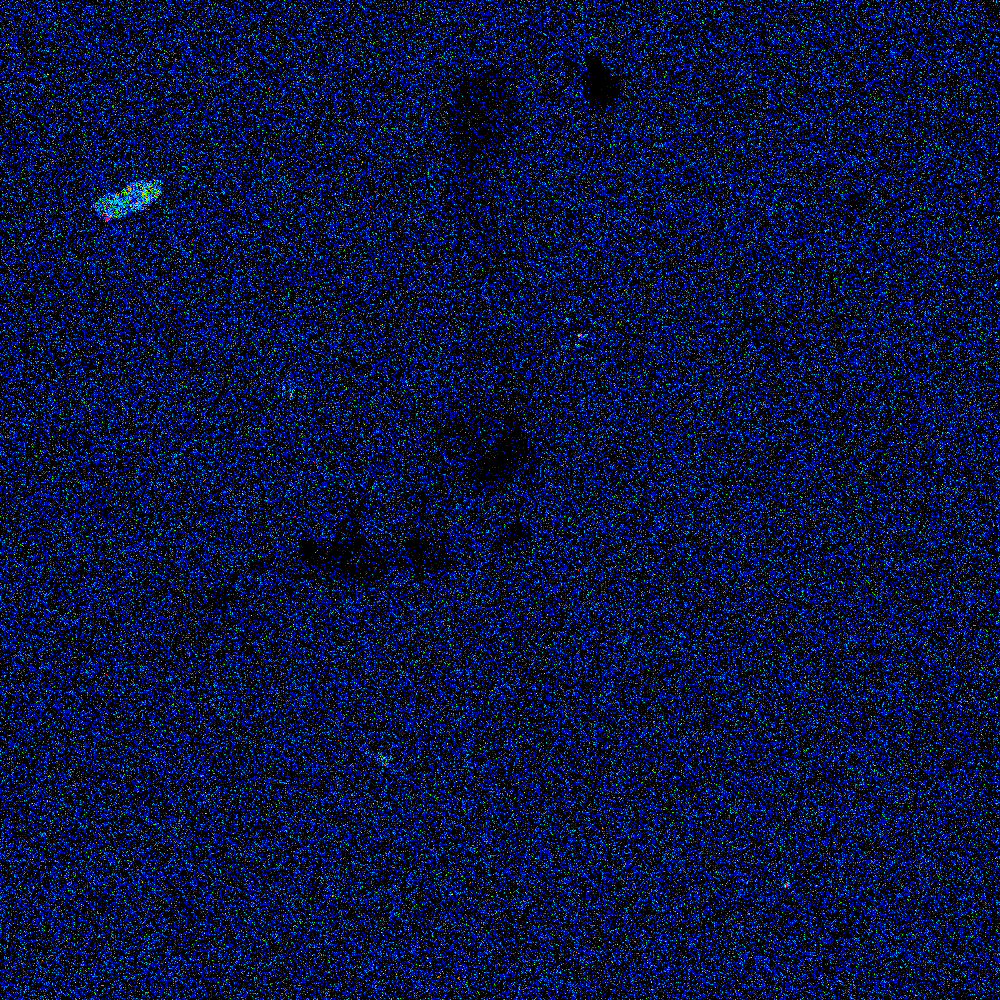

Supplement: Supplementary file 1 [file materials-12-02019-s001.zip › Raw Data for MDPI Repository/CHA (SSZ-13)/WDS/Cl.tif]

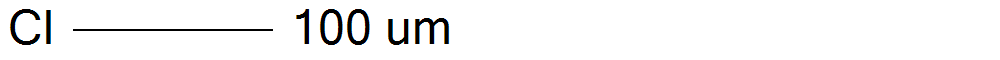

Supplement: Supplementary file 1 [file materials-12-02019-s001.zip › Raw Data for MDPI Repository/CHA (SSZ-13)/WDS/Cl_marker.tif]

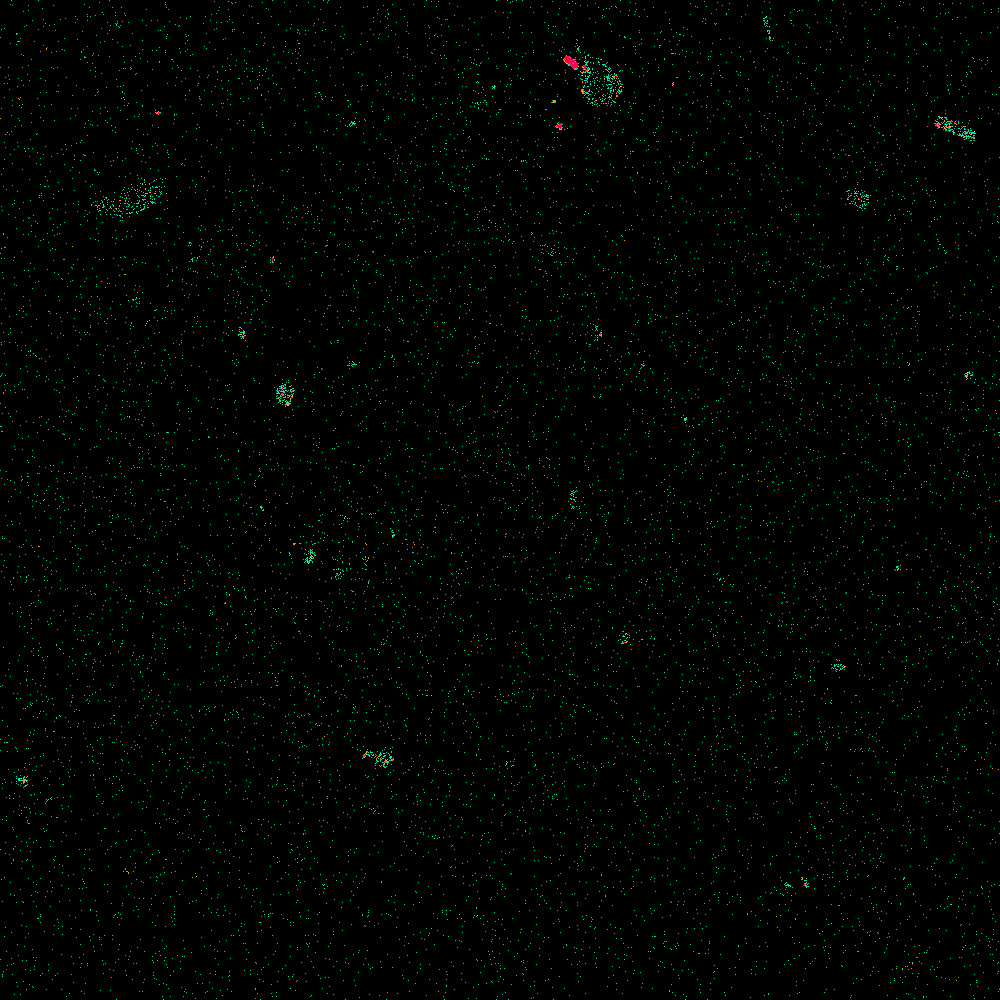

Supplement: Supplementary file 1 [file materials-12-02019-s001.zip › Raw Data for MDPI Repository/CHA (SSZ-13)/WDS/Ca.tif]

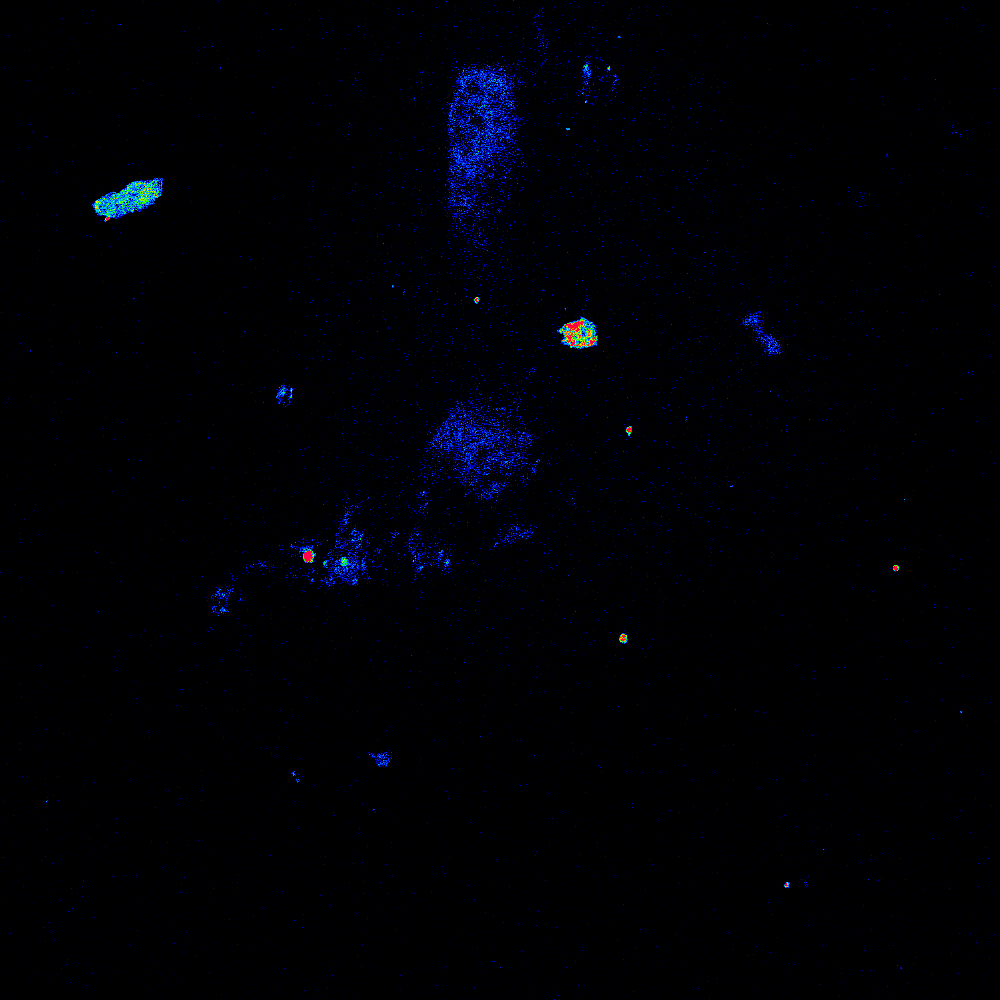

Supplement: Supplementary file 1 [file materials-12-02019-s001.zip › Raw Data for MDPI Repository/CHA (SSZ-13)/WDS/Na.tif]

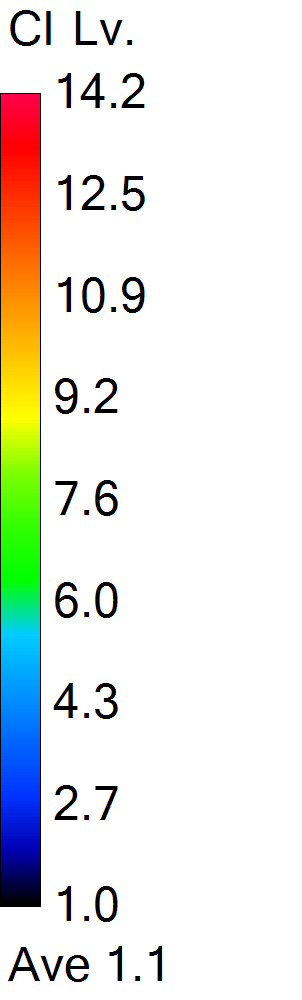

Supplement: Supplementary file 1 [file materials-12-02019-s001.zip › Raw Data for MDPI Repository/CHA (SSZ-13)/WDS/Cl_2_color.tif]

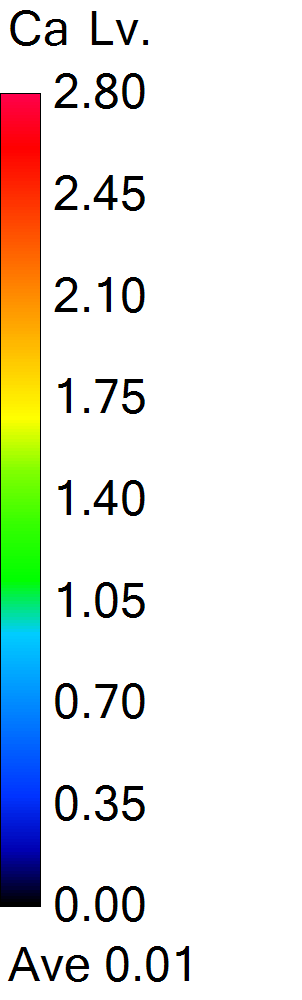

Supplement: Supplementary file 1 [file materials-12-02019-s001.zip › Raw Data for MDPI Repository/CHA (SSZ-13)/WDS/Ca_color.tif]

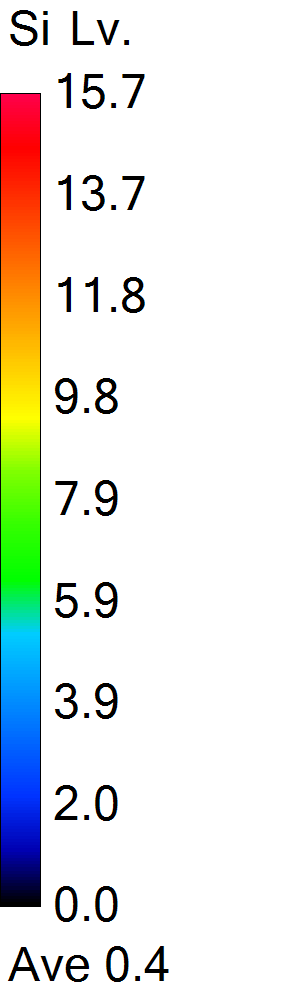

Supplement: Supplementary file 1 [file materials-12-02019-s001.zip › Raw Data for MDPI Repository/CHA (SSZ-13)/WDS/Si_color.tif]

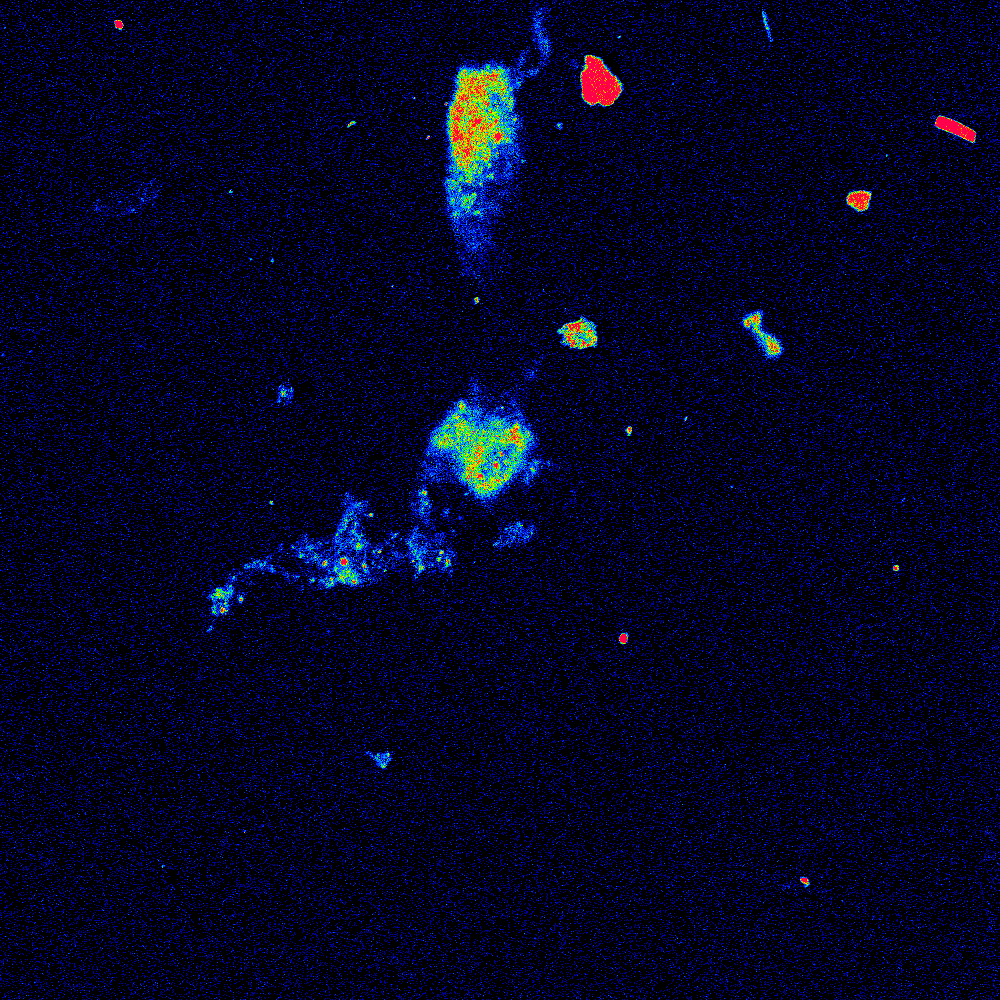

Supplement: Supplementary file 1 [file materials-12-02019-s001.zip › Raw Data for MDPI Repository/CHA (SSZ-13)/WDS/Si.tif]

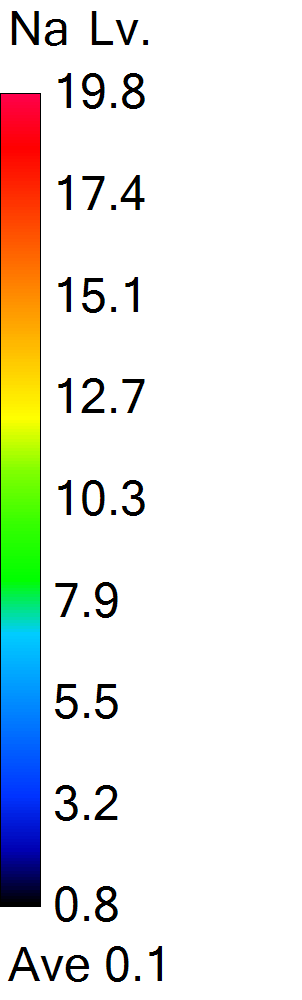

Supplement: Supplementary file 1 [file materials-12-02019-s001.zip › Raw Data for MDPI Repository/CHA (SSZ-13)/WDS/Na_color.tif]

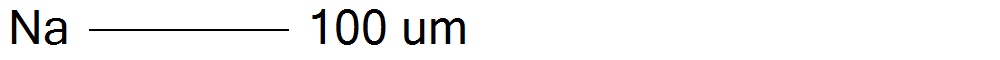

Supplement: Supplementary file 1 [file materials-12-02019-s001.zip › Raw Data for MDPI Repository/CHA (SSZ-13)/WDS/Na_marker.tif]

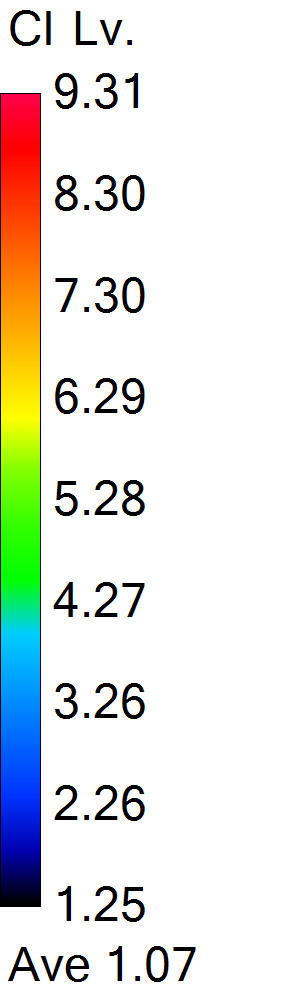

Supplement: Supplementary file 1 [file materials-12-02019-s001.zip › Raw Data for MDPI Repository/CHA (SSZ-13)/WDS/Cl_color.tif]

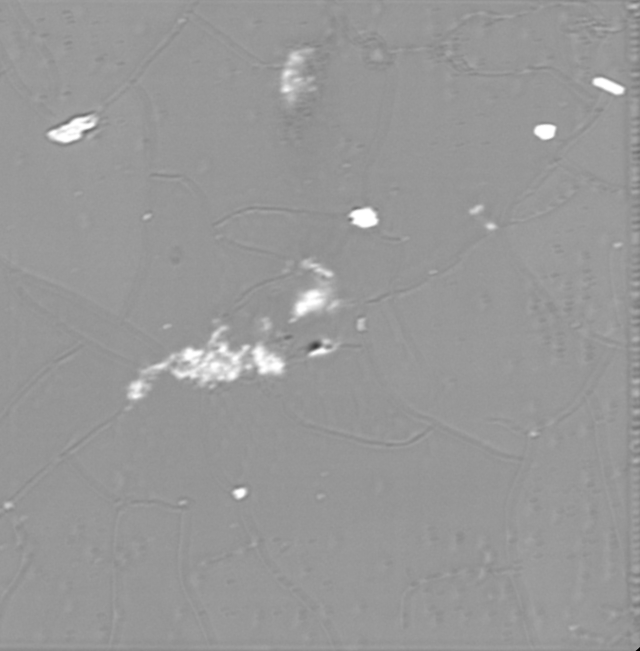

Supplement: Supplementary file 1 [file materials-12-02019-s001.zip › Raw Data for MDPI Repository/CHA (SSZ-13)/SSZ-13_2_cropped.tif]

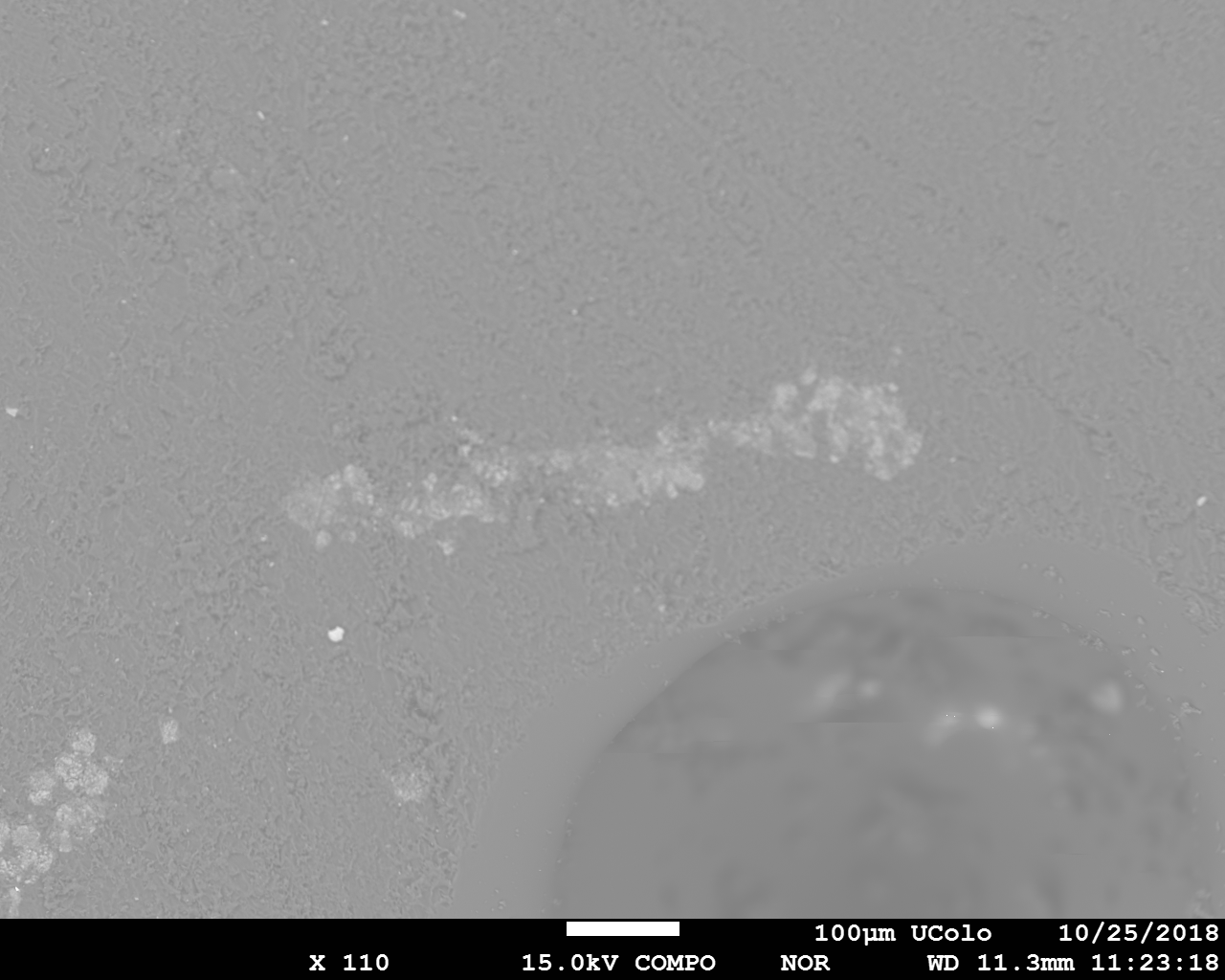

Supplement: Supplementary file 1 [file materials-12-02019-s001.zip › Raw Data for MDPI Repository/CHA (SSZ-13)/SSZ-13 Control.tif]

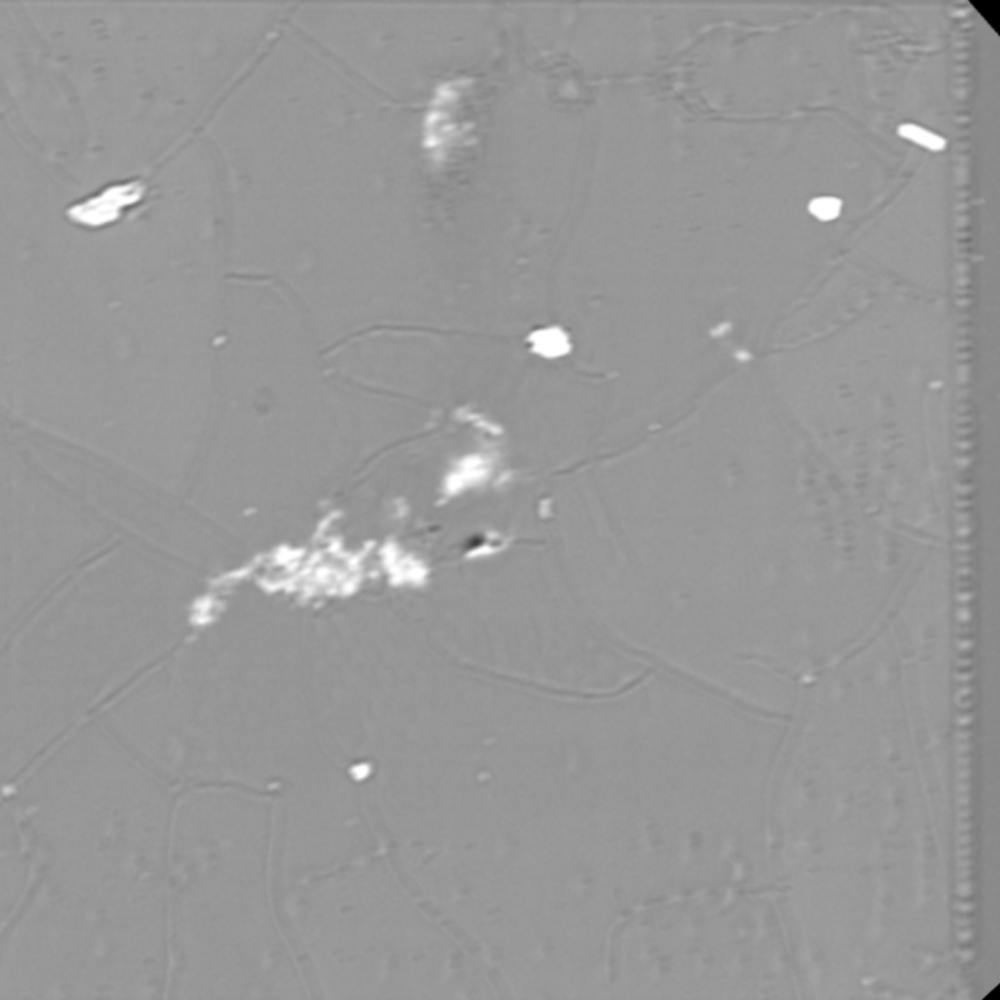

Supplement: Supplementary file 1 [file materials-12-02019-s001.zip › Raw Data for MDPI Repository/CHA (SSZ-13)/SSZ-13_2_cropped_very cropped.tif]

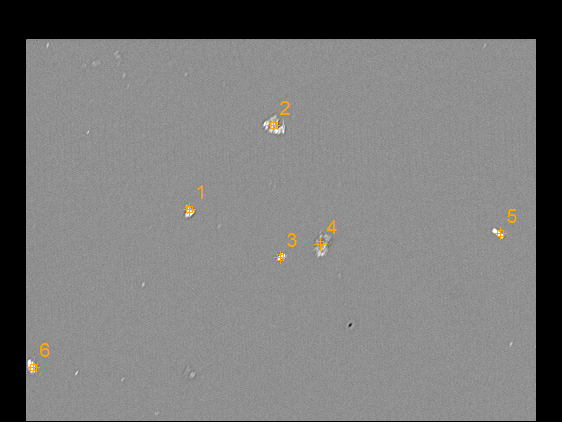

Supplement: Supplementary file 1 [file materials-12-02019-s001.zip › Raw Data for MDPI Repository/CHA (SSZ-13)/SSZ-13_7/Base(27).psref.24bit.tif]

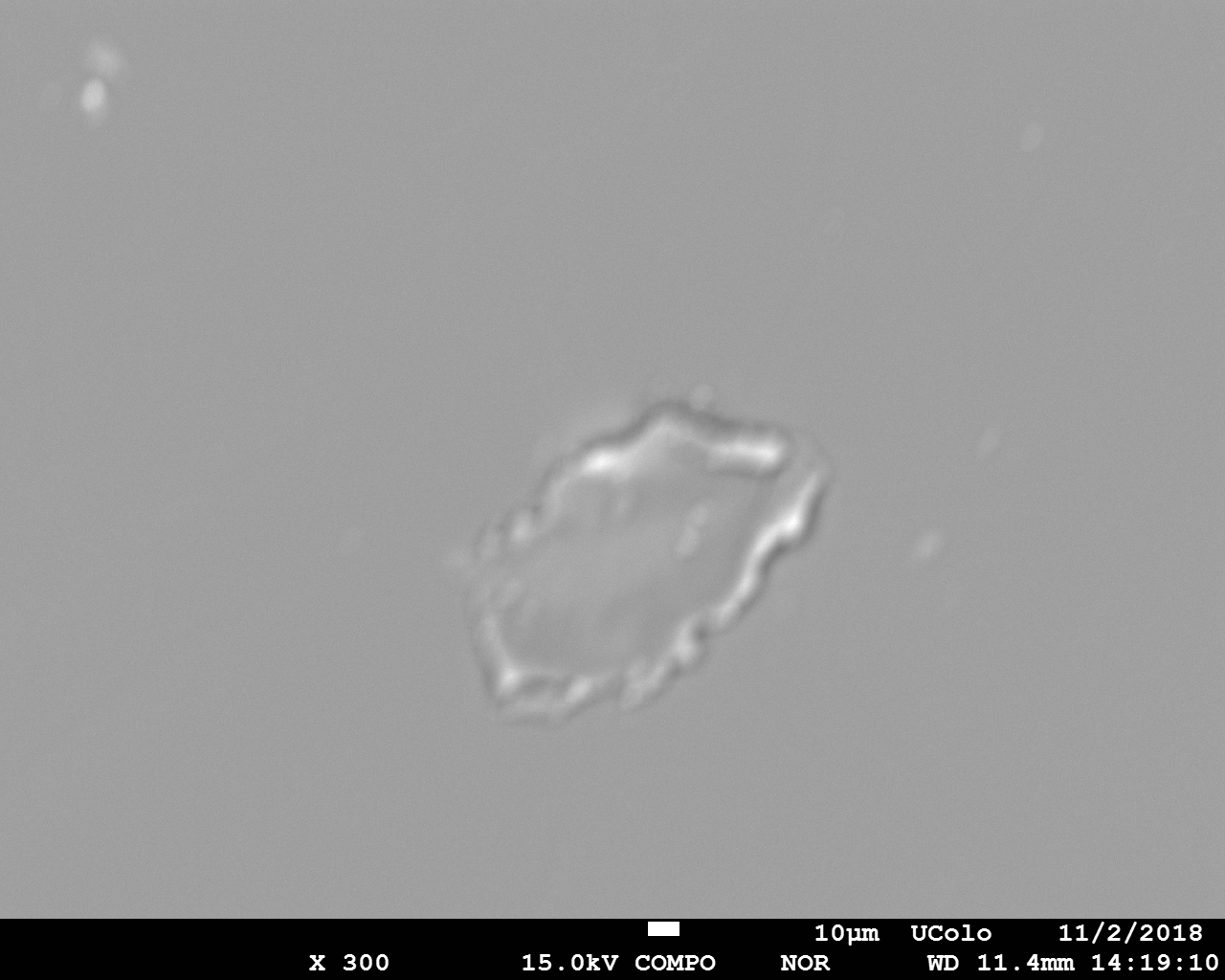

Supplement: Supplementary file 1 [file materials-12-02019-s001.zip › Raw Data for MDPI Repository/CHA (SSZ-13)/SSZ-13_3.tif]

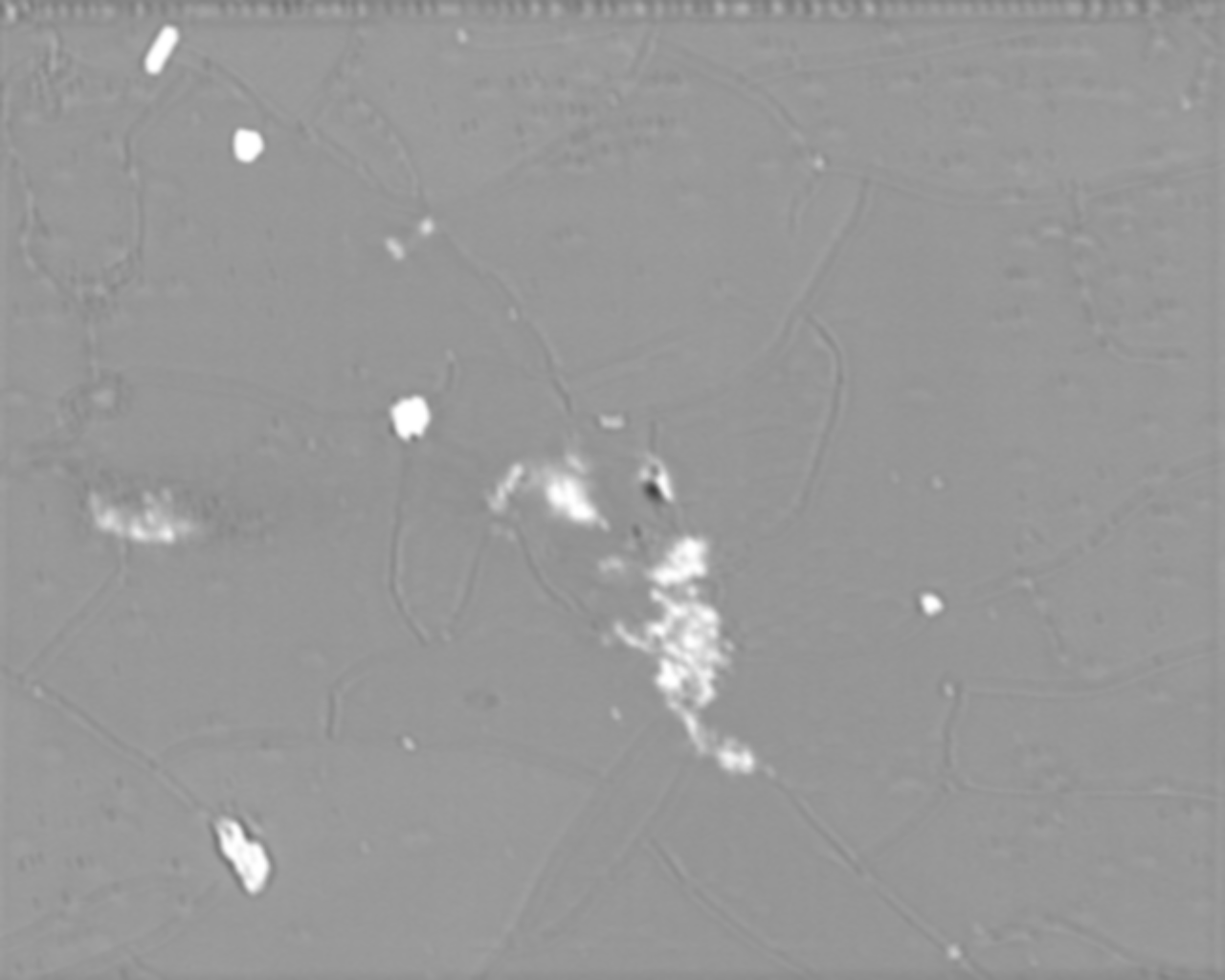

Supplement: Supplementary file 1 [file materials-12-02019-s001.zip › Raw Data for MDPI Repository/CHA (SSZ-13)/SSZ-13_2-2.tif]

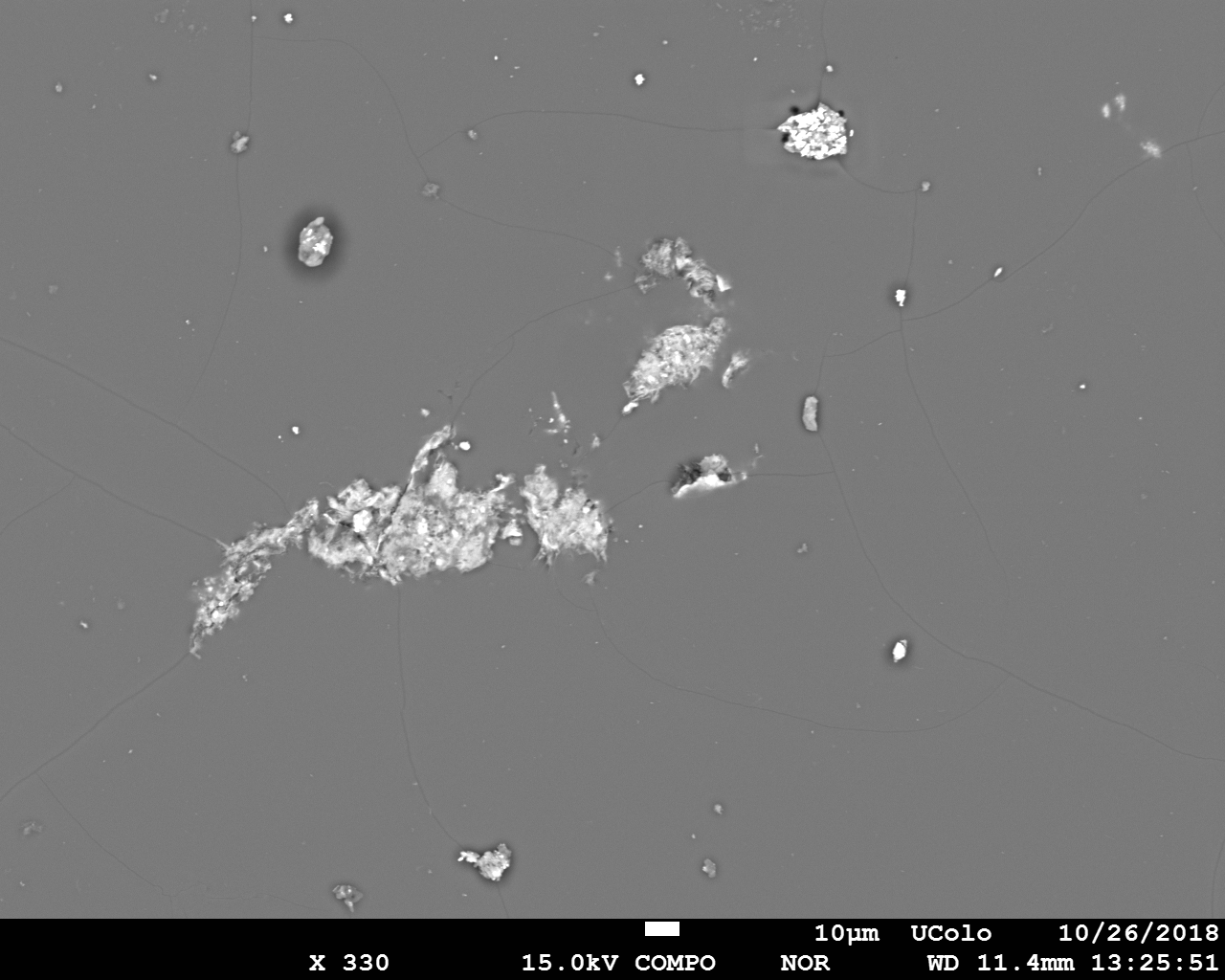

Supplement: Supplementary file 1 [file materials-12-02019-s001.zip › Raw Data for MDPI Repository/CHA (SSZ-13)/SSZ-13 Exposed.tif]

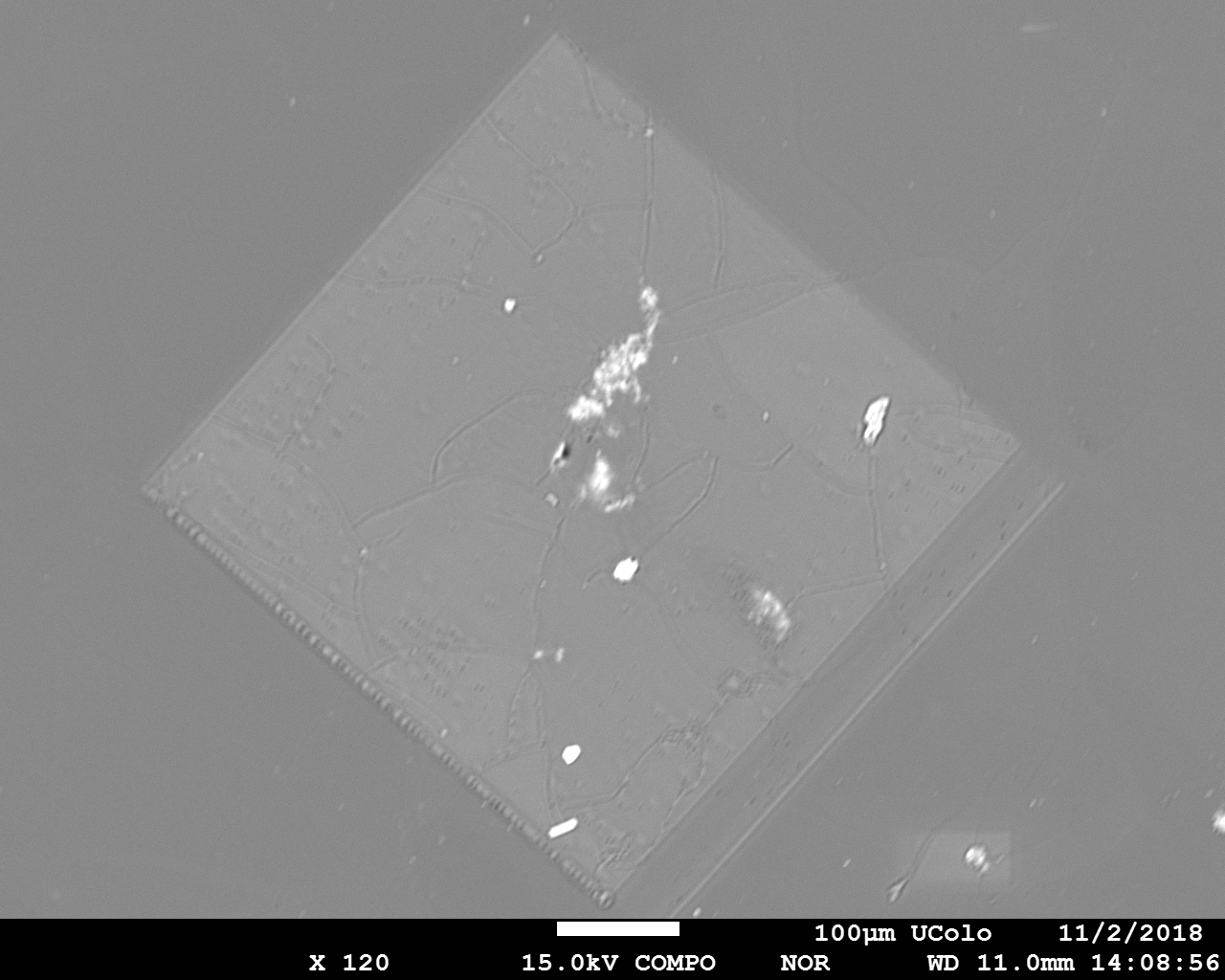

Supplement: Supplementary file 1 [file materials-12-02019-s001.zip › Raw Data for MDPI Repository/CHA (SSZ-13)/SSZ-13_2.tif]

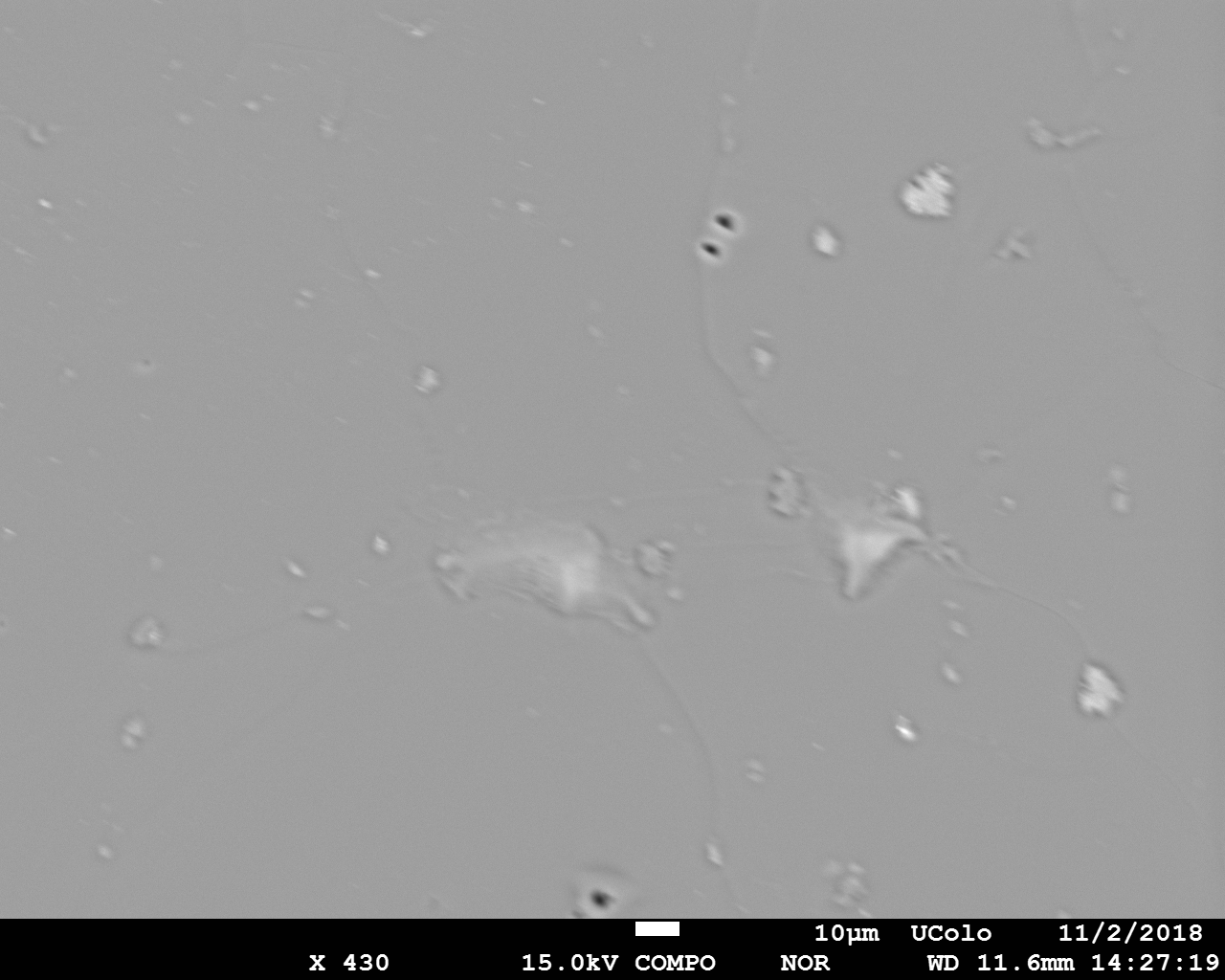

Supplement: Supplementary file 1 [file materials-12-02019-s001.zip › Raw Data for MDPI Repository/CHA (SSZ-13)/SSZ-13_5.tif]

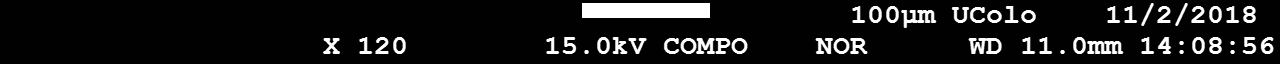

Supplement: Supplementary file 1 [file materials-12-02019-s001.zip › Raw Data for MDPI Repository/CHA (SSZ-13)/SSZ-13_2-1_Scale.tif]

## Slide 1
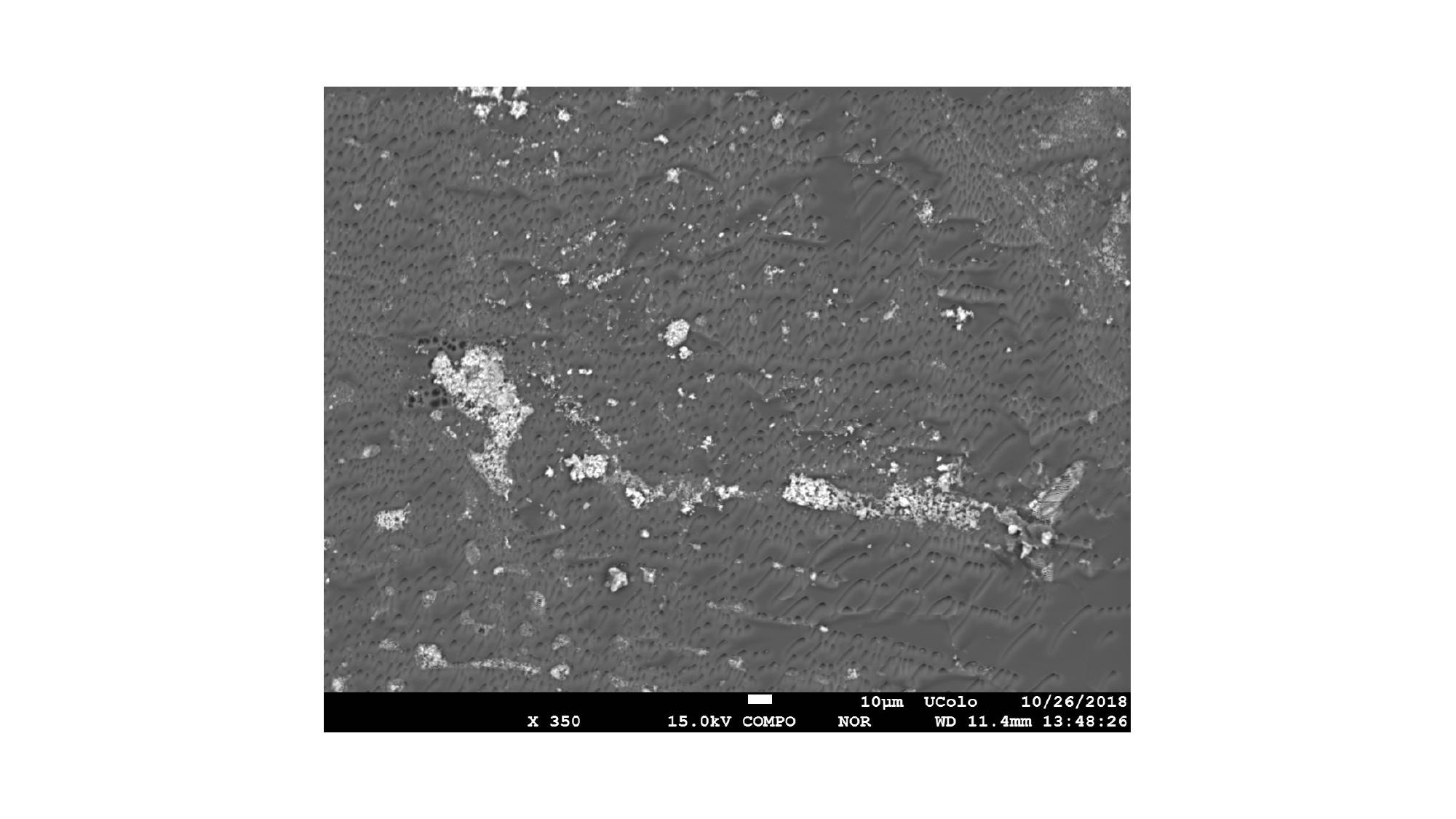

## Slide 2
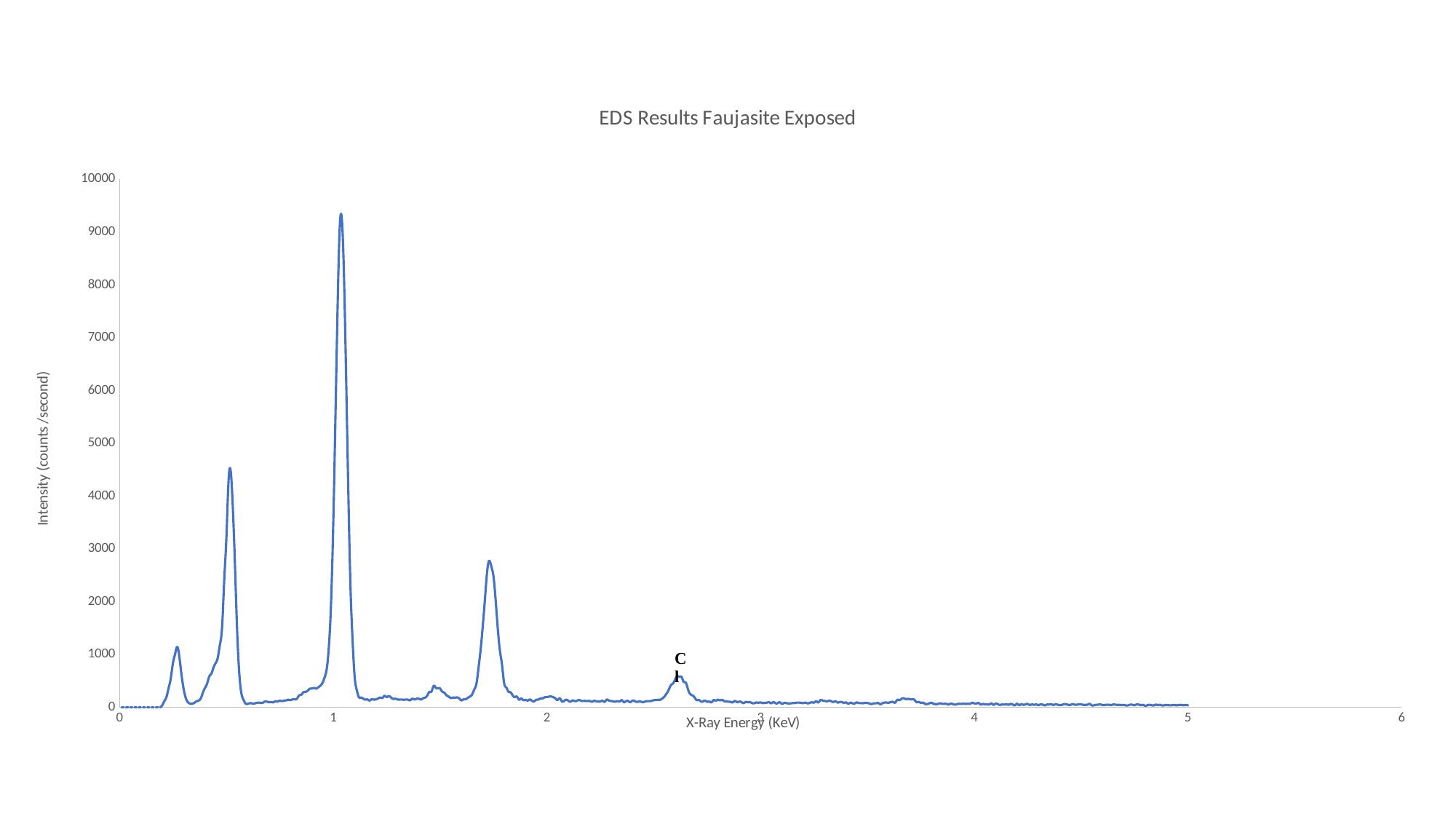

### Chart: EDS Results Faujasite Exposed
| Category | |
|---|---|

Supplement: Supplementary file 1 [file materials-12-02019-s001.zip › Raw Data for MDPI Repository/FAU/EDS/EDS Results.pptx]

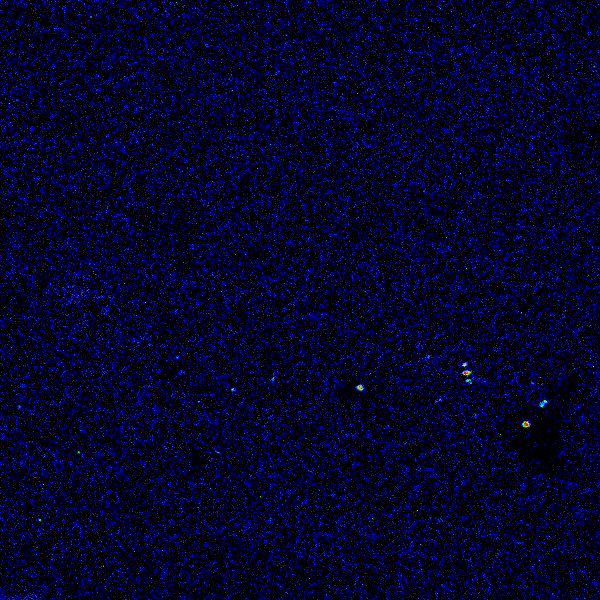

Supplement: Supplementary file 1 [file materials-12-02019-s001.zip › Raw Data for MDPI Repository/FAU/WDS/Cl_2.tif]

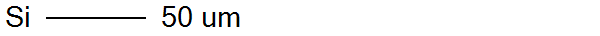

Supplement: Supplementary file 1 [file materials-12-02019-s001.zip › Raw Data for MDPI Repository/FAU/WDS/Si_marker.tif]

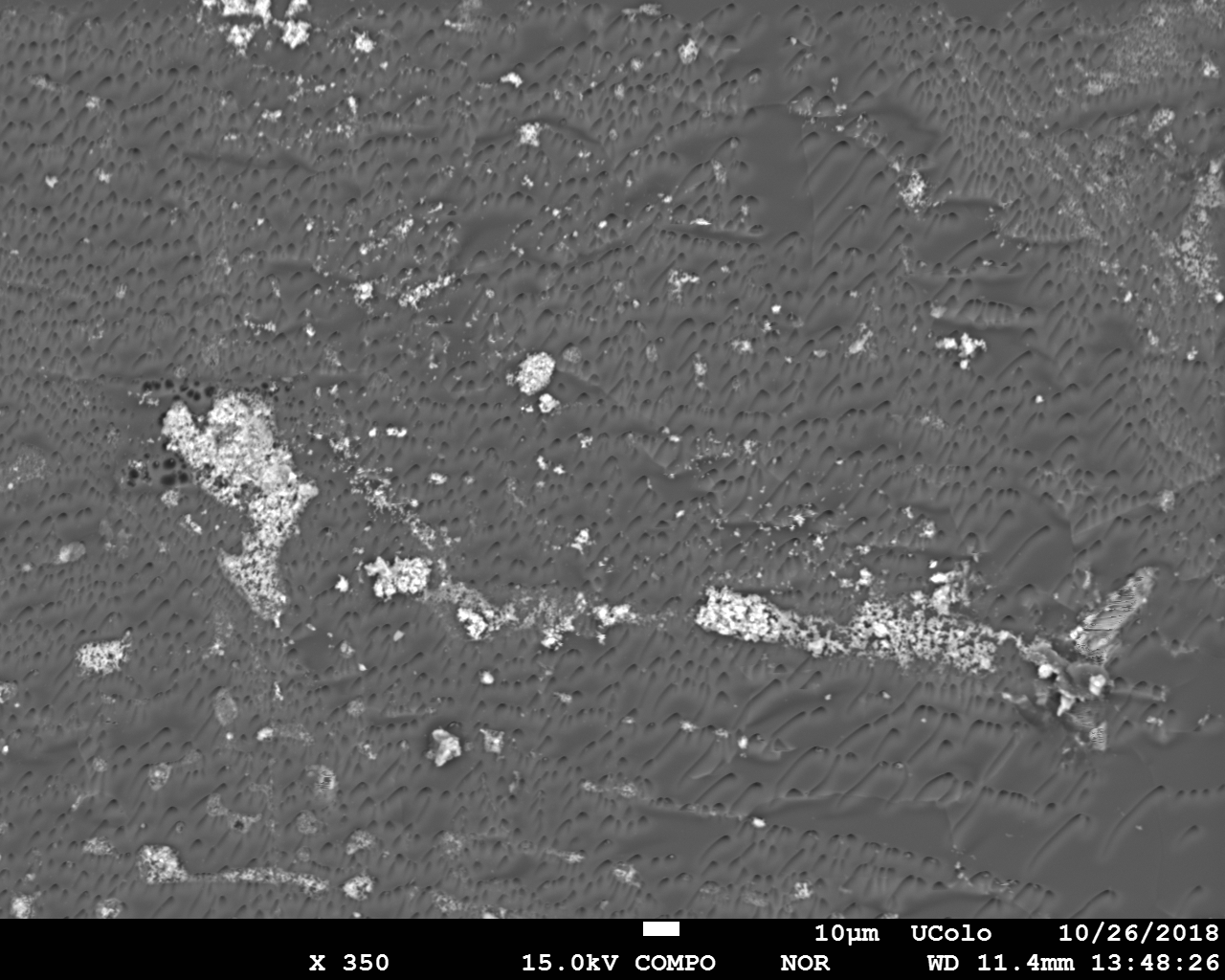

Supplement: Supplementary file 1 [file materials-12-02019-s001.zip › Raw Data for MDPI Repository/FAU/WDS/Faujasite Exposed.tif]

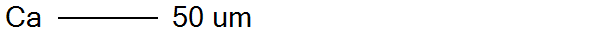

Supplement: Supplementary file 1 [file materials-12-02019-s001.zip › Raw Data for MDPI Repository/FAU/WDS/Ca_marker.tif]

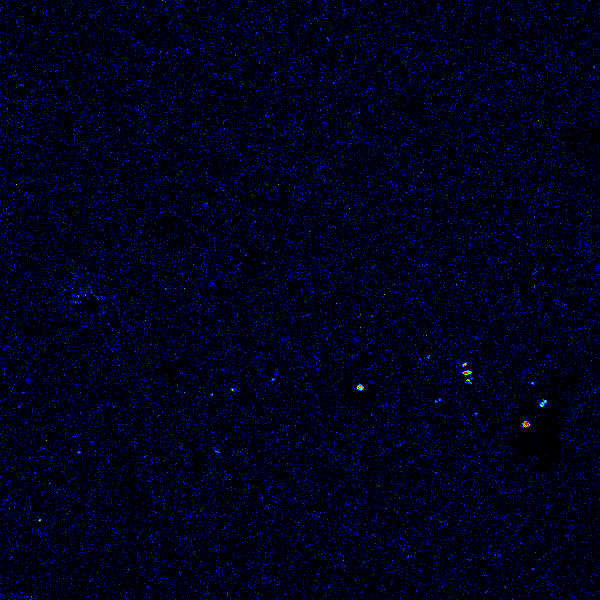

Supplement: Supplementary file 1 [file materials-12-02019-s001.zip › Raw Data for MDPI Repository/FAU/WDS/Cl.tif]

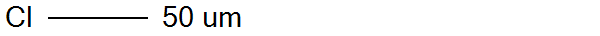

Supplement: Supplementary file 1 [file materials-12-02019-s001.zip › Raw Data for MDPI Repository/FAU/WDS/Cl_marker.tif]

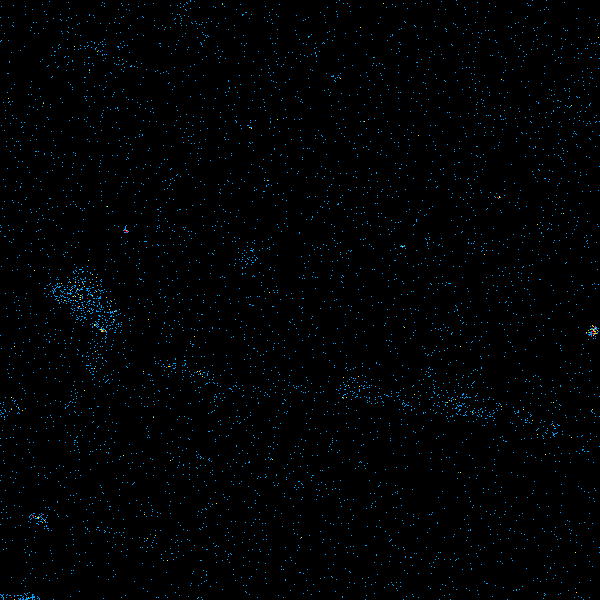

Supplement: Supplementary file 1 [file materials-12-02019-s001.zip › Raw Data for MDPI Repository/FAU/WDS/Ca.tif]

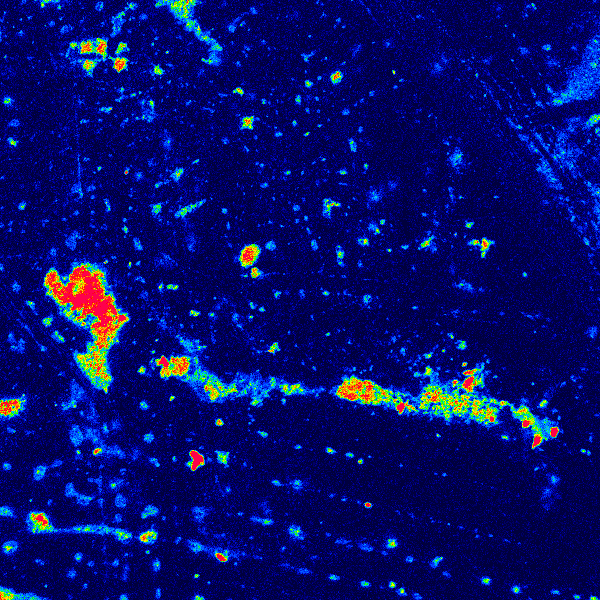

Supplement: Supplementary file 1 [file materials-12-02019-s001.zip › Raw Data for MDPI Repository/FAU/WDS/Na.tif]

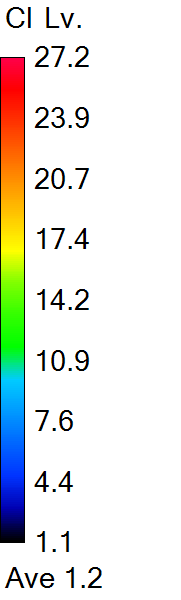

Supplement: Supplementary file 1 [file materials-12-02019-s001.zip › Raw Data for MDPI Repository/FAU/WDS/Cl_2_color.tif]

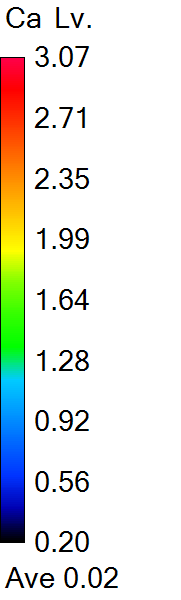

Supplement: Supplementary file 1 [file materials-12-02019-s001.zip › Raw Data for MDPI Repository/FAU/WDS/Ca_color.tif]

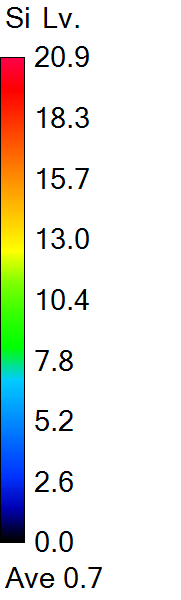

Supplement: Supplementary file 1 [file materials-12-02019-s001.zip › Raw Data for MDPI Repository/FAU/WDS/Si_color.tif]

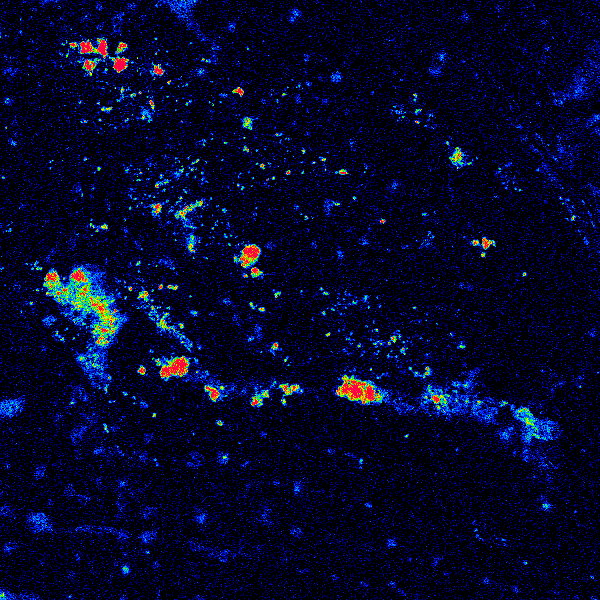

Supplement: Supplementary file 1 [file materials-12-02019-s001.zip › Raw Data for MDPI Repository/FAU/WDS/Si.tif]

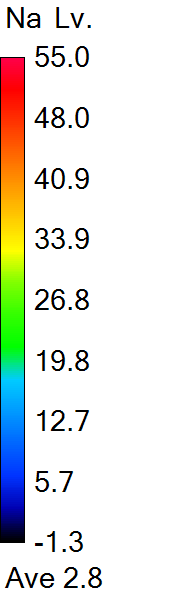

Supplement: Supplementary file 1 [file materials-12-02019-s001.zip › Raw Data for MDPI Repository/FAU/WDS/Na_color.tif]

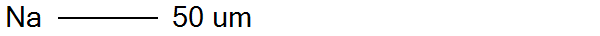

Supplement: Supplementary file 1 [file materials-12-02019-s001.zip › Raw Data for MDPI Repository/FAU/WDS/Na_marker.tif]

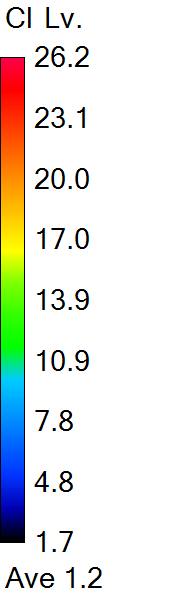

Supplement: Supplementary file 1 [file materials-12-02019-s001.zip › Raw Data for MDPI Repository/FAU/WDS/Cl_color.tif]
